# Supplementary material for: Maternal emotional and physical intimate partner violence and early child development: investigating mediators in a cross-sectional study in a South African birth cohort
Source: BMJ Open. 2021 Oct 28;11(10):e046829. doi: 10.1136/bmjopen-2020-046829 (PMC8557296; doi:10.1136/bmjopen-2020-046829)
Supplement: Supplementary data [file bmjopen-2020-046829supp001.pdf]

Supplemental Table 1. Comparison of psychosocial, demographic and clinical data between children included in complete case developmental models and those who are not.

	In Development models	Not in Development model	p-value
Intimate Partner Violence (IPV)			
Emotional IPV score, median (IQR)	0 (0, 0)	0 (0, 0)	0.669
Physical IPV score, median (IQR)	0 (0, 0)	0 (0, 0)	0.213
Sexual IPV score, median (IQR)	0 (0, 0)	0 (0, 0)	0.160.
Sociodemographics			
Maternal education (did not complete secondary)	393 (63)	302 (58)	0.132
Mother employed	225 (41)	99 (51)	0.022*
Mother married/partnered	268 (49)	99 (51)	0.718
Household Income			
<R5000	445 (73)	122 (63)	0.671
>R5000	164 (27)	47 (24)	
Recruitment site: TC Newman	274 (44)	235 (45)	0.586
Physical variables			
Birth weight z-score, median (IQR)	-0.59 (-1.34, 0.16)	-0.51 (-1.22, -0.00)	0.744
Male sex	321 (51)	269 (52)	0.774
HIV exposed	153 (24)	94 (18)	0.010*
Length for age z-scores, median (IQR)	-1.10 (-1.80, -0.34)	-1.03 (-1.85, -0.28)	0.846
Weight for age z-scores, median (IQR)	-0.31 (-1.10, 0.43)	-0.24 (-1.01, 0.40)	0.6682
Weight for length z-scores, median (IQR)	0.28 (-0.48, 1.13)	0.43 (-0.40, 1.09)	0.7542
Psychosocial factors			
Depression score, median (IQR)	0 (0, 3)	0 (0, 5)	0.415
Alcohol dependence score, median (IQR)	0 (0,0)	0 (0,0)	0.068

Data are presented as n(%) or median IQR). Birthweight and HIV exposure collected at birth; all other variables collected at 2 years of child age. Abbreviation: IPV=intimate partner violence.
*p<0.05; **p<0.001

Supplemental Table 2. Univariate associations between intimate partner violence, covariates and proposed mediators and composite scores for developmental domains in the multiple-imputation analysis sample.

	Composite Cognitive<br>Unadjusted<br>Coefficient (95% CI)	Composite Language<br>Unadjusted<br>Coefficient (95% CI)	Composite Motor<br>Unadjusted<br>Coefficient (95% CI)
Intimate Partner Violence (IPV)			
Emotional IPV score	-0.33 (-0.60, -0.06)*	-0.34 (-0.68, -0.01)*	-0.51 (-0.86, -0.16)*
Physical IPV score	-0.19 (-0.43, 0.05)	-0.20 (-0.50, 0.11)	-0.39 (-0.70, -0.09)*
Sexual IPV score	-0.13 (-0.98, 0.71)	-0.16 (-1.36, 1.04)	-0.41 (-1.58, 0.75)
Sociodemographics			
Maternal education (not completed secondary)	-1.31 (-2.69, 0.07)	-3.20 (-4.94, -1.45)**	-1.45 (-3.30, 0.40)
Mother employed	0.48 (-1.02, 1.99)	0.13 (-1.81, 2.07)	0.41 (-1.55, 2.38)
Mother married/partnered	-0.59 (-2.07, 0.89)	0.26 (-1.64, 2.16)	0.15 (-1.88, 2.18)
Household Income			
>R5000	0.73 (-0.92, 2.38)	1.00 (-1.14, 3.13)	1.96 (-0.28, 4.20)
TC Newman	1.20 (-0.15, 2.54)	3.12 (1.41, 4.84)**	0.89 (-0.91, 2.68)
Physical variables			
Birth weight z-score	0.44 (-0.15, 1.03)	0.68 (-0.07, 1.42)	0.58 (-0.21, 1.36)
Male	-1.67 (-3.01, -0.33)*	-4.07 (-5.77, -2.37)**	-1.70 (-3.49, 0.09)
Child age (months)	-0.95 (-2.24, 0.33)	-0.12 (-1.77, 1.53)	-2.81 (-4.50, -1.11)*
HIV exposed	-1.70 (-3.29, -0.10)*	-3.77 (-5.82, -1.72)**	-0.59 (-2.72, 1.54)
Length for age z-score	0.92 (0.33, 1.52)*	0.67 (-0.09, 1.42)	0.26 (-0.54, 1.06)
Weight for age z-score	0.84 (0.26, 1.43)*	0.64 (-0.10, 1.37)	0.82 (0.04, 1.60)*
Weight for length z-score	0.50 (-0.08, 1.08)	0.39 (-0.34, 1.11)	0.88 (0.10, 1.66)*
Proposed Psychosocial mediators			
Depression score	-0.02 (-0.12, 0.08)	-0.06 (-0.18, 0.07)	-0.11 (-0.24, 0.02)
Alcohol dependence score	-0.08 (-0.19, 0.03)	-0.03 (-0.17, 0.11)	0.03 (-0.11, 0.16)

Birthweight and HIV exposure collected at birth; all other variables collected at 2 years of child age. IPV sub-types, depression and alcohol dependence were included as continuous variables in all models.
Abbreviation: IPV= intimate partner violence.
*p<0.05, **p<0.001

Supplemental Table 3. Multivariable linear regression demonstrating the association of intimate partner violence with composite scores for developmental domains in the multiple-imputation analysis sample.

	Cognitive scores	Language scores	Motor scores	
	Adjusted#<br>Coefficient (95% CI)	Adjusted^<br>Coefficient (95% CI)	Adjusted+<br>Coefficient (95% CI)	Adjusted+<br>Coefficient (95% CI)
Emotional IPV score	-0.30 (-0.57, -0.03)*	-0.35 (-0.68, -0.01)	-0.52 (-0.87, -0.17)*	
Physical IPV score				-0.39 (-0.70, -0.09)*

Adjusted models were run only where IPV sub-type was associated (p<0.05) with outcome explored in bivariate analyses. For cognitive and language scores, only emotional IPV was associated in bivariate analyses and therefore run in multivariable models. Due to collinearity, emotional and physical IPV were run in separate multivariable models for motor development. Adjusted models were run including same covariates as the complete case analysis, as noted below. IPV sub-types, depression and alcohol dependence were included as continuous variables in all models.
Abbreviations: IPV=intimate partner violence.
*p<0.05; **p<0.001
#Adjusted for child sex, HIV exposure, length-for-age z-scores, weight-for-age z-scores and weight-for-length z-scores at 24 months.
^Adjusted for maternal education, recruitment site, weight-for-age z-score at birth, child sex and HIV exposure.
+Adjusted for child age at assessment and weight-for-length z-scores at 24 months.

Supplemental Table 4. Univariate associations between proposed mediators and intimate partner violence in complete case sample.

	**Emotional IPV** Coefficient (95% CI)	**Physical IPV** Coefficient (95% CI)
Depression score	1.01 (0.80, 1.22)**	0.68 (0.49, 0.87)**
Alcohol dependence score	0.34 (0.13, 0.56)*	0.30 (0.12, 0.49)*

IPV sub-types, depression and alcohol dependence were included as continuous variables in all models.
Abbreviation: IPV = intimate partner violence.
*p<0.05, **p<0.001

Supplemental Table 5. Univariate and adjusted associations between proposed mediators and developmental outcomes in complete case sample.

		**Cognitive scores**[#] Coefficient (95% CI)	**Language scores^** Coefficient (95% CI)	**Motor scores**[+] Coefficient (95% CI)
Univariate	Depression	-0.02 (-0.11, 0.08)	-0.06 (-0.18, 0.06)	-0.12 (-0.25, 0.01)
	Alcohol	-0.07 (-0.17, 0.03)	-0.03 (-0.16, 0.11)	0.01 (-0.13, 0.15)
Adjusted	Depression	0.02 (-0.08, 0.13)	-0.05 (-0.18, 0.07)	-0.04 (-0.19, 0.10)
	Alcohol	-0.06 (-0.17, 0.05)	0.01 (-0.12, 0.14)	0.05 (-0.10, 0.20)

Notes: Adjusted associations between proposed mediators and developmental domains were run using linear regression. Depression and alcohol were run separately in adjusted regression models. IPV sub-types, depression and alcohol dependence were included as continuous variables in all models.
Abbreviation: IPV = intimate partner violence.
#Adjusted for intimate partner violence, child sex, HIV exposure, length-for-age z-scores, weight-for-age z-scores and weight-for-length z-scores at 24 months.
^Adjusted for intimate partner violence, maternal education, recruitment site, weight-for-age z-score at birth, child sex and HIV exposure.
+Adjusted for intimate partner violence, child age at assessment and weight-for-length z-scores at 24 months.
