# Supplementary material for: Maternal emotional and physical intimate partner violence and early child development: investigating mediators in a cross-sectional study in a South African birth cohort
Source: BMJ Open. 2021 Oct 28;11(10):e046829. doi: 10.1136/bmjopen-2020-046829 (PMC8557296; doi:10.1136/bmjopen-2020-046829)
Supplement: Author's manuscript [file bmjopen-2020-046829.draft_revisions.pdf]

BMJ Open is committed to open peer review. As part of this commitment we make the peer review history of every article we publish publicly available.

When an article is published we post the peer reviewers' comments and the authors' responses online. We also post the versions of the paper that were used during peer review. These are the versions that the peer review comments apply to.

The versions of the paper that follow are the versions that were submitted during the peer review process. They are not the versions of record or the final published versions. They should not be cited or distributed as the published version of this manuscript.

BMJ Open is an open access journal and the full, final, typeset and author-corrected version of record of the manuscript is available on our site with no access controls, subscription charges or pay-per-view fees (http://bmjopen.bmj.com).

If you have any questions on BMJ Open's open peer review process please email info.bmjopen@bmj.com

BMJ Open

Maternal emotional and physical intimate partner violence and early child development: investigating mediators in a cross sectional study in a South African birth cohort.

Journal:	*BMJ Open*
Manuscript ID	bmjopen-2020-046829
Article Type:	Original research
Date Submitted by the Author:	12-Nov-2020
Complete List of Authors:	Barnett, Whitney; University of Cape Town Faculty of Health Sciences, Department of Paediatrics and Child Health & South African Medical Research Council Unit on Child & Adolescent Health<br>Halligan, Sarah; University of Bath, Department of Psychology; University of Cape Town Faculty of Health Sciences, Department of Psychiatry and Mental Health & South African Medical Research Council Unit on Risk and Resilience in Mental Disorders<br>Wedderburn, Catherine; London School of Hygiene & Tropical Medicine, Department of Clinical Research; University of Cape Town, Department of Paediatrics and Child Health & South African Medical Research Council Unit on Child & Adolescent Health<br>MacGinty, Rae; University of Cape Town Faculty of Health Sciences, Department of Paediatrics and Child Health & South African Medical Research Council Unit on Child & Adolescent Health<br>Hoffman, Nadia; University of Cape Town Faculty of Health Sciences, Department of Psychiatry and Mental Health & South African Medical Research Council Unit on Risk and Resilience in Mental Disorders<br>Zar, Heather; University of Cape Town Faculty of Health Sciences, Department of Paediatrics and Child Health & South African Medical Research Council Unit on Child & Adolescent Health<br>Stein, Dan; University of Cape Town Faculty of Health Sciences, Department of Psychiatry and Mental Health & South African Medical Research Council Unit on Risk and Resilience in Mental Disorders; University of Cape Town Faculty of Health Sciences, Neuroscience Institute<br>Donald, Kirsten; University of Cape Town Faculty of Health Sciences, Division of Developmental Paediatrics, Department of Paediatrics and Child Health; University of Cape Town Faculty of Health Sciences, Neuroscience Institute
Keywords:	Developmental neurology & neurodisability < PAEDIATRICS, PUBLIC HEALTH, TRAUMA MANAGEMENT

SCHOLARONE™
Manuscripts

I, the Submitting Author has the right to grant and does grant on behalf of all authors of the Work (as defined in the below author licence), an exclusive licence and/or a non-exclusive licence for contributions from authors who are: i) UK Crown employees; ii) where BMJ has agreed a CC-BY licence shall apply, and/or iii) in accordance with the terms applicable for US Federal Government officers or employees acting as part of their official duties; on a worldwide, perpetual, irrevocable, royalty-free basis to BMJ Publishing Group Ltd ("BMJ") its licensees and where the relevant Journal is co-owned by BMJ to the co-owners of the Journal, to publish the Work in this journal and any other BMJ products and to exploit all rights, as set out in our licence.

The Submitting Author accepts and understands that any supply made under these terms is made by BMJ to the Submitting Author unless you are acting as an employee on behalf of your employer or a postgraduate student of an affiliated institution which is paying any applicable article publishing charge ("APC") for Open Access articles. Where the Submitting Author wishes to make the Work available on an Open Access basis (and intends to pay the relevant APC), the terms of reuse of such Open Access shall be governed by a Creative Commons licence – details of these licences and which Creative Commons licence will apply to this Work are set out in our licence referred to above.

Other than as permitted in any relevant BMJ Author's Self Archiving Policies, I confirm this Work has not been accepted for publication elsewhere, is not being considered for publication elsewhere and does not duplicate material already published. I confirm all authors consent to publication of this Work and authorise the granting of this licence.

For peer review only

Maternal emotional and physical intimate partner violence and early child development: investigating mediators in a cross sectional study in a South African birth cohort.

Whitney Barnett[a], Sarah L Halligan[b,c], Catherine J Wedderburn[a,d], Rae MacGinty[a], Nadia Hoffman[c], Heather J. Zar[a], Dan J. Stein[c,e], Kirsten A Donald[e,f]

[a] Department of Paediatrics and Child Health & South African Medical Research Council Unit on Child & Adolescent Health, University of Cape Town, South Africa; [b] Department of Psychology, University of Bath, United Kingdom; [c] Department of Psychiatry and Mental Health & South African Medical Research Council Unit on Risk and Resilience in Mental Disorders, University of Cape Town, South Africa; [d] Department of Clinical Research, London School of Hygiene & Tropical Medicine, London, UK; [e] Neuroscience Institute, University of Cape Town, South Africa [f] Division of Developmental Paediatrics, Department of Paediatrics & Child Health, University of Cape Town, South Africa

Corresponding author:
Whitney Barnett
Department of Paediatrics and Child Health
Unit B11, REACH Center
Red Cross War Memorial Children's Hospital
Cape Town, South Africa
barnett.whitney@gmail.com
Tel: +27 (0) 790432604

Key words: toddler development, intimate partner violence, LMIC, IPV, child development

Word count: 4,080

Funding

The study was funded by the Bill and Melinda Gates Foundation [OPP 1017641]. Additional support for HJZ and DJS is from the SA MRC. Additional aspects of the work reported here are supported by an Academy of Medical Sciences Newton Advanced Fellowship (NAF002\1001), funded by the UK Government's Newton Fund, by the UK MRC (MR/T002816/1) and by the US Brain and Behaviour Foundation Independent Investigator grant (24467). WB is supported by the SAMRC, through its Division of Research Capacity Development under the Bongani Mayosi National Health Scholars programme. CJW was supported by the Wellcome Trust through a Research Training Fellowship [203525/Z/16/Z].

Acknowledgements

We thank the study staff in Paarl, the study data team and lab teams, the clinical and administrative staff of the Western Cape Government Health Department at Paarl Hospital and at the clinics for support of the study. We thank the families and children who participated in this study.

For peer review only

Abstract

Objectives: This study investigated associations between recent maternal intimate partner violence (emotional, physical and sexual) and child development at 2 years as well as whether maternal depression or alcohol use mediated these relationships.

Design: Cross sectional study nested in a South African birth cohort.

Setting: Two primary care clinics in Paarl, South Africa.

Participants: 626 mother-child pairs; inclusion criteria for maternal antenatal enrolment were clinic attendance and remaining in the study area for at least 1 year; women were excluded if a minor.

Primary outcome measures: Child cognitive, language and motor development composite scores. These were assessed using the Bayley Scales of Infant and Toddler Development, 3rd edition (BSID-III).

Results: Emotional IPV was associated with lower cognitive (β=-0.32; 95%CI -0.60,-0.04), language (β=-0.36; 95%CI -0.69,-0.01) or motor composite scores (β=-0.58; 95%CI -0.95,-0.20) in children at 2 years of age. Physical IPV was associated with lower motor scores (β=-0.42; 95%CI -0.75,-0.09) at 2 years. Sexual IPV was unrelated to developmental outcomes, possibly due to low prevalence. Neither recent maternal depression nor alcohol use were shown to mediate the relationship between IPV and developmental outcomes.

Conclusions: Interventions to reduce maternal IPV, including emotional IPV, and early-life interventions for infants and toddlers are needed to promote optimal child development.

Strengths and limitations of this study

- This study investigates the impact of multiple types of IPV (emotional, physical and sexual) on developmental outcomes in very young children in a high-risk setting.
- Understanding the factors underlying associations between IPV and poor child development is critical to better inform interventions and policy, thus we investigated both maternal postnatal depression and alcohol use as potential mediators of IPV-child development relationships in this cohort.
- The current study used cross sectional data and therefore did not investigate longitudinal exposures and cannot assert causality, however, results highlight the potential importance of IPV exposure during a developmentally sensitive period.

For peer review only

Background

Intimate partner violence (IPV) is one of the most common forms of violence against women. The World Health Organization's multicountry study on violence against women found high lifetime prevalence of physical IPV (up to 61%), sexual IPV (up to 59%) and emotional IPV (up to 75%).[1] The epidemic of IPV is particularly marked in low-and-middle-income countries (LMICs), including South Africa, where prevalence is amongst the highest globally.[2,3] Children in households with IPV are often witnesses and are at increased risk for abuse or neglect.[4] The United Nations estimates that each year between 133–275 million children witness violence between primary caregivers.[5] Exposure to violence in the home is linked to many adverse health outcomes in children, including developmental, psychological and behavioural impairments.[6-8] Importantly, these often impact health and development beyond childhood, having negative effects that extend into adolescence and adulthood.[6,9,10] However, there remain several gaps in the existing literature.

Relatively little research has examined associations between the presence of IPV and poorer cognitive, language and motor development in infancy and very early childhood[11-13] though it is a critical developmental period that can have significant implications for long-term health.[14] Where data are available, exposure to IPV in the first two years of life has been associated with increased risk of developmental delay.[12,13] Healthy development during the first years of life is vital, given its impact on academic performance and mental health across the lifespan.[10,14] Indeed, a longitudinal study through 8 years found that exposure to interpersonal trauma (maltreatment and/or witnessing IPV) in the first two years of life was associated with the poorest cognitive outcomes compared to other periods in childhood.[15]

For peer review only

Research focusing on child development and IPV often narrowly defines IPV as only physical or sexual abuse, though emotional IPV has been linked to similar or increased rates of ill-health, both physical and mental, compared to physical IPV.[16-19] Additionally, the majority of studies, particularly in school-aged children, have been done in high income countries (HICs), though LMICs have a higher burden of IPV-associated risk factors, such as community violence, trauma, substance use disorders and mental illness and limited support networks. South Africa in particular has among the highest burdens of interpersonal violence in the world.[20] The roots of high levels of violence can be linked to South Africa's colonial and apartheid past, including the impacts of systemic racism, impoverishment, migrant labour, gender norms and constructions of masculinity.[21] Zembe and colleagues investigated social risk factors and relationship power inequity and IPV in a South African sample, finding high acceptability of violence in intimate relationships as well as gender norms supporting aggressive masculinity and subservient femininity, particularly in historically marginalized groups such as those included in the present study.[22]

Lastly, a key gap in the existing literature examining associations between IPV exposure and early life child developmental outcomes is investigation of potential mediators in this relationship, particularly in LMIC settings. Several potential mechanisms underlying the relationship between IPV and child development have been proposed. For instance, maternal depression as well as alcohol use have been separately linked to both IPV and development outcomes,[13,19] potentially compromising maternal-child attachment, impacting maternal autonomy and caregiving, diverting financial resources in the home and may increase the risk of child maltreatment or abuse.[23] Alcohol is one of the most commonly misused substances, with extremely high prevalence of alcohol use disorders in South Africa,[24] and maternal use postnatally is

For peer review only

associated with poorer child development and academic performance.[25-27] Maternal depression has been linked to insecure mother-child attachment;[28] less sensitive and responsive caregiving,[29] and reduced health care service utilization[30] adversely impacting care and stimulation needed for healthy development. Although there is evidence linking proposed mediators to both IPV and early-life development, extremely few have formally investigated potential explanatory factors.[31]

This study aims to explore associations between IPV sub-types (emotional, physical and sexual) and child development (cognitive, language and motor) at two years of age and to investigate potential mechanisms underlying this relationship. The current study addresses three key gaps in the literature, specifically by (i) investigating associations between IPV and development in very young children (ii) investigating the differential impact of sub-types of IPV and (iii) investigating maternal depression or alcohol use as potential mediators in these relationships. Given the high prevalence of IPV in low resource settings, understanding the significant impact IPV exposure may have on young children during this developmentally sensitive period and improved understanding of possible pathways underlying these relationships is important for improving child potential globally.[11]

Methods

The parent study is a longitudinal, population-based birth cohort study following mother–child dyads from pregnancy through childhood.[32-34] The study is located in a periurban area in South Africa, characterised by low socioeconomic status (SES) and psychosocial risk factors. There is a free primary health care system, that includes antenatal and child health services. The current cross sectional analysis uses data from three study visits occurring at or near 2 years of child age.

Participants

Pregnant women were enrolled from March 2012 to March 2015. Women were enrolled in their second trimester, between 20 and 28 weeks' gestation at two public sector primary healthcare clinics, one serving a predominantly mixed-ancestry population and the other serving a predominantly Black African population. Our intention in using the historic terms of *Black African* and *mixed ancestry* is not meant to reify these terms, but to contribute to the literature on ongoing health disparities. Inclusion criteria were: (1) pregnant women attending one of the two study clinics; (2) aged 18 years or older; and (3) intending to remain in the study area for at least 1 year.

Ethical considerations

Ethical approval was obtained from the Faculty of Health Sciences Research Ethics Committee, University of Cape Town (401/2009) and the Western Cape Provincial Research committee. Mothers gave written informed consent at enrolment and were re-consented annually thereafter. Study staff were trained in ethical conduct of violence and mental health research, including confidentiality, and identification and management of risk. Interviews were conducted privately, data were de-identified and only accessible to study staff to ensure confidentiality. Where significant mental health issues or safety concerns were identified, staff referred participants to appropriate care or social services (including support services for IPV, substance abuse and mental health issues).

For peer review only

Clinical and sociodemographic data

Sociodemographic information was collected from the mother at a visit two years after their child's birth, using a standardized questionnaire adapted from items included in the South African Stress and Health Study.[35] Maternal education (any secondary versus completed secondary), employment and partnership status (single or married/marriage-like relationship) and household income [<R,1000/month (60USD), R1,000 to R5000 (350USD) or > R5,000/month] were self-reported at 2 years postpartum. Maternal HIV status during pregnancy was confirmed by routine testing during antenatal care. All HIV-exposed children were tested per the Western Cape prevention of mother-to-child transmission guidelines.[36] Birthweight and postnatal anthropometric measurements were collected by trained staff at birth and 2 years, as has been described.[37]

Intimate partner violence

Recent maternal IPV was measured at 2 years postpartum using the Intimate Partner Violence Questionnaire (IPVQ), a 12-item inventory adapted from the WHO multicountry study[38] and the Women's Health Study in Zimbabwe.[39] The IPVQ assessed recent (past-year) exposure to emotional (4 of 12 questionnaire items), physical (5 of 12 items), and sexual abuse (3 of 12 items). Partner behavior indicating emotional IPV included having been insulted or made to feel bad, having been humiliated in front of others, intentionally scared or intimidated or threatened with physical harm. Physical IPV included being slapped, pushed, shoved, hit with an object, beaten or choked. Sexual IPV exposure was classified based on having been forced to have sex, afraid not to have sex or forced to do something sexual which was degrading or humiliating. Mothers were asked about frequency of exposure to partner

For peer review only - http://bmjopen.bmj.com/site/about/guidelines.xhtml

behavior ("never", "once", "a few times" or "many times"). Items were then summed to create scores for each IPV sub-type with higher scores indicating greater severity and frequency of exposure; exposure was defined as a score >1 indicating more than an isolated incident within each subtype. Scoring guidelines were devised for the purposes of this study, and were based on prior work in South Africa.[40]

Proposed mediating variables

Recent maternal depression was measured using the Beck Depression Inventory (BDI), a validated and reliable screening tool for depressive symptoms at 2 years post-partum.[41,42] The BDI-II comprises 21 items, which assesses severity of symptoms of major depression on a severity scale from 0 (absence of symptoms) to 3 (severe). Individual items are summed to obtain a total score, with higher scores indicating more severe depressive symptoms. A cut off score of ≥20 was used to indicate participants with probable depression.[43] The Alcohol, Smoking and Substance Involvement Screen Test (ASSIST) was used to measure current maternal alcohol-related risk at 2 years of child age.[44] Alcohol related items were summed to create a score for alcohol-related problems with higher scores indicating greater substance-related risk. We used published guidelines for the ASSIST to classify alcohol scores into three categories for descriptive purposes: low/no risk (0-10), moderate risk (11-26) and high risk (>26), with the likelihood of alcohol dependence.[45]

Bayley Scales of Infant and Toddler Development, 3rd edition (BSID-III)

The Bayley Scales of Infant and Toddler Development, third edition (BSID-III)[46] was used to measure toddler neurodevelopment. It is widely used to assess development in children ages 2 to 42 months and has been validated for use in South

For peer review only - http://bmjopen.bmj.com/site/about/guidelines.xhtml

Africa.[47] Assessments for neurodevelopment were done at 2 years of child age by trained physiotherapists and occupational therapists supervised by a paediatric neurodevelopmental specialist.[34] Quality control and monitoring processes were implemented to ensure accuracy. Assessments were conducted with language prompts in the child's preferred language and assessors alternated between sites and assessed a similar number of children. In the current study, composite scores for cognition, language and motor development were used. The composite scores are calculated using a normal US population, and are standardised to have a mean of 100 and standard deviation of 15. Composite scores are strongly correlated to raw scores in the current study and allow comparison across domains. Continuous composite scores as well as categorical outcomes are reported. Categories were defined as 'delay' (<2 standard deviations below mean), 'suboptimal development' (<1 standard deviation below mean) and 'no delay'.

Patient and public involvement

Investigators have established close relationships with key stakeholders including Western Cape Government Health Department and community members. Participants were not involved in the design or recruitment of the DCHS. However, participant experience and satisfaction have been assessed at multiple time points.[48] Participant feedback about study experience, information provided and study procedures have been used ongoing to reassess study protocols and to make alternations to improve study experience and participant satisfaction. Study findings are routinely fed back to the community and the Department of Health clinical and administrative staff.

Data analysis

The analyses were conducted using STATA 15 (StataCorp Inc., College Station, Texas, USA). Demographic, clinical, psychosocial and child development data were described using median (interquartile range (IQR)) for continuous data or number (%) for categorical data. Developmental data were compared by child sex using Pearson's Chi-square test for categorical data and the Wilcoxon Rank Sum test for continuous data.

Mean composite scores were calculated for the Bayley-III for cognitive, language and motor domains. Birthweight was converted to z-scores based on child sex and gestational age using the INTERGROWTH-21st standards [49]. Postnatal weight-for-age z-scores (WFAZ), length-for-age z-scores (LFAZ) and weight-for-length z-scores (WFHZ) were calculated using weight and length measurements at 24 months, using Anthro software (WHO, Geneva, Switzerland) [50]. These as well as emotional, physical and sexual IPV and proposed mediator variables were included as continuous scores in all analyses.

We examined associations using a complete case analysis sample. Bivariate linear regression analyses were used to investigate the relationship between developmental domains, clinical and demographic variables, proposed mediators and IPV sub-types. Regression coefficients with 95% confidence intervals (CIs) are reported. Multivariable linear regression models were then run for each of the developmental (composite cognitive, language and motor scores) outcomes, including significant univariable coefficients ($p<0.05$). Continuous scores were used for exposure, mediator and outcome data in multivariable models. Where multiple IPV sub-types were significant in bivariate regression, adjusted models were run for each separately, as these were highly correlated. Hypothesized mediators of the relationship

For peer review only

between IPV and early life development (current maternal depression and alcohol use) were investigated. Criteria for formal investigation of potential mediators were 1) their association with developmental outcomes, using a cut-off level of $p<0.05$, in final adjusted models (path b) and 2) where associations between IPV and the hypothesized mediator were significant (path a).

In addition to the analysis of observations with complete data, we examined associations after imputing missing data for covariates and developmental outcomes using multiple imputation with chained equations (MICE). We assumed covariate data were missing at random and performed 30 imputations. Results from complete case and imputed datasets produced similar conclusions. Results based on imputed data are presented in Supplemental Tables. To explore potential selection bias, we assessed the characteristics of mother-child dyads with complete and missing data, using Pearson's Chi-squared and Wilcoxon Rank Sum tests to compare covariate distributions between groups.

Results

Of 1143 study births, 154 (13.5%) children were lost to follow up before reaching two years of age primarily due to moving out of the study area or being uncontactable or unavailable for follow up visits. A further 255 (25.8%) did not complete a BSID-III assessment due to non-attendance on the day or moving out of the study area. Data for the current study was collected across three visits at approximately 2 years of child age: a clinical visit for growth and sociodemographic data collection; a psychosocial visit for maternal depression and substance use data and a visit assessing child development. A total of 626 children who completed the BSID-III assessment and had IPV exposure and covariate data were included in the final complete case dataset.

For peer review only

Comparing the characteristics of dyads with complete and missing exposure or outcome data, mothers with missing data were less likely to be HIV infected (p=0.01) and less likely to be employed (p=0.02, Supplemental Table 1).

Clinical, demographic, and psychosocial characteristics at two years are presented in Table 1. This sample was characterized by low maternal employment (41%), low maternal education (37% completed secondary education), low household income (only 27% earning more than R5,000 per month [approximately USD 350]). Almost half of mothers were married or cohabiting (49%) and 24% of children were HIV exposed with only 2 children HIV-infected. A minority of mothers experienced psychosocial risk factors or substance use, including emotional IPV (16%), physical IPV (17%) and sexual IPV (4%), alcohol (10% moderate/high risk), and depression (11%), Table 1.

Developmental outcomes

A total of 28/625 (4%) children were categorised as having cognitive delay (<2 standard deviations below mean), 48/598 (8%) with language delay and 7/592 (1%) with motor delay. Only language differed significantly by child sex, with males having a higher prevalence of delay compared to females (10% versus 5%; p value=0.001) and lower composite scores (p<0.001), Table 2.

Intimate partner violence and developmental outcomes

At 24 months, bivariate analysis showed that emotional IPV was associated with lower composite cognitive scores (β=-0.32; 95% CI -0.58,-0.06), lower composite language scores (β=-0.35; 95% CI -0.69,-0.01) and lower composite motor scores (β=-0.58; 95% CI -0.93,-0.24). Physical IPV was associated with lower composite motor

view only

scores (β=-0.42; 95% CI -0.73,-0.11). Sexual IPV was not associated with any composite developmental outcomes, Table 3.

Multivariable regression models were run to examine associations between IPV and child developmental outcomes, adjusting for key potential confounding factors. IPV sub-types were run separately in adjusted models due to collinearity and only where significant in bivariate analyses. In adjusted models, the results held, recent emotional IPV was associated with reduced scores across all developmental outcomes, specifically with lower composite cognitive scores (β -0.32; 95% CI -0.60,-0.04), language (β -0.35; 95% CI -0.69, -0.01) and motor scores (β -0.58; 95% CI -0.95, -0.20). Physical IPV was associated with lower motor scores (β -0.42; 95% CI -0.75, -0.09), Table 4. Associations using the imputed dataset were similar for key exposures (IPV) and outcomes (developmental), Supplemental Table 2 & 3.

Hypothesized mediators

Current maternal depression and alcohol use were explored as potential mediators in the relationship between recent IPV sub-types and developmental outcomes. In correlational analyses, both maternal depression and alcohol use were positively associated with emotional as well as physical IPV (Supplemental Table 4). However, when linear regressions were run investigating associations between mediators and developmental outcomes, adjusted for potential confounding factors, we found no evidence of associations between proposed mediators and each developmental outcome (cognitive, language, motor), Supplemental Table 5. Therefore, formal mediation analyses were not done.

Discussion

For peer review only

Our results show that recent maternal emotional IPV was associated with lower cognitive, language and motor scores and physical IPV with lower motor scores in children at 24 months. Further, we investigated the potential role of current maternal depression or alcohol use in this relationship. There was insufficient evidence that current maternal depression or alcohol use were explanatory factors in the relationship between IPV and child development at 2 years of age.

We found associations between emotional IPV and each developmental outcome investigated, which are novel findings in such young children. Though there is limited research in this age group, and existing studies use differing definitions of IPV, a recent study pooling data from 11 LMICs investigating pre-school development (36-59 months) found associations between all IPV sub-types and developmental outcomes.[51] Where investigated in a younger sample, studies have found a link between IPV and early life development. However, few investigated IPV sub-types as separate risk factors for poor development. For example, a US study, investigating the impact of IPV on developmental outcomes at two years reported an increased risk of language and neurological delay in infants and toddlers, however this was not analysed by IPV sub-type but rather a composite measure of emotional, physical and sexual IPV.[12] A Tanzanian study through three years of age found both maternal lifetime physical and lifetime sexual IPV to be associated with reduced motor skills, expressive and receptive communication and cognitive development.[13] Emotional IPV was not included in their IPV measure. One study that did investigate emotional IPV separately as a risk factor for cognitive and language delay, only found associations with postnatal physical IPV and only in unadjusted analyses. The limited sample size (n=72) may have reduced statistical power to detect an association.[52] In the current study, sexual IPV was not associated in unadjusted or adjusted analyses with the outcomes investigated, but this

For peer review only - http://bmjopen.bmj.com/site/about/guidelines.xhtml

is likely due to insufficient power to detect an association given the relatively low prevalence of recent sexual IPV in our study population (4%). In the current study, maternal emotional IPV as compared to physical IPV was more consistently associated with poorer developmental outcomes. These results indicate that emotional IPV may carry a unique risk for poor child developmental outcomes in early life in our setting.

Given the high prevalence of emotional IPV and that it is often not as readily recognised or included in research, intervention or policy efforts aimed at prevention of IPV, it is critical to better understand its distinct impact on child health outcomes. Our findings suggest that adverse outcomes and their association with maternal IPV may be underestimated when IPV exposure is not considered broadly and inclusive of emotional IPV, a potentially significant gap in the literature. Emotional abuse in a current or past intimate relationships is increasingly being recognized as a unique contributing factor to maternal ill-health outcomes.[17-19] Specifically, emotional IPV has been linked to poor mental health, post traumatic stress disorder and poorer physical health, sometimes above that of physical IPV.[18,53] Psychological control or abuse by a partner may uniquely impact maternal efficacy, independence and diminish maternal power within the family, which may affect her ability to provide nutritious food, a secure environment or to have positive parenting practices, all of which could lead to increased risk of negative outcomes for her children. These factors may be particularly influential for health outcomes in settings such as South Africa where entrenched gender norms and a high acceptability of violence contribute to high levels of IPV.[22]

Our investigation of potential mediators found little evidence to support that current maternal depression symptoms or reported alcohol use were explanatory factors for the impact of IPV on child development at two years. In the parent study we have previously found that IPV is linked to longitudinal maternal depression[19] as well as

For peer review only

hazardous alcohol use in pregnancy.[54] In the current study, we found that IPV was associated with depression and alcohol use at 2 years. However, these did not appear to be associated with development at 2 years. Recent maternal depression or alcohol use may compromise the quality of parenting or parent-child attachment, may divert household financial resources and decrease maternal care-seeking behaviours, which in turn may compromise child development. Though there is much literature linking maternal substance use to adverse child outcomes, some studies that have investigated its impact on parenting or secure attachment have found mixed results.[55,56] For example, a study assessing maternal substance use and quality of caregiving found no differences between substance abusing and non-substance abusing mothers and caregiving or maternal-child attachment at 12 months.[55] Similarly, studies investigating maternal depression and child development have shown mixed findings.[13,57,58] A Tanzanian study investigating depression and toddler development, found that depression was associated with receptive language and motor development but not cognitive or expressive language.[13] Another study found that maternal depression at 7 weeks postpartum impacted development at 3 months but this relationship attenuated by 6 months.[57] Our findings may be influenced by relatively low levels of probable clinical depression (11%) or moderate-high alcohol-related risk (10%), diluting possible associations. We found no evidence that recent maternal depression or alcohol use influenced the relationship between IPV and early life development; these findings may illustrate the complexity of the relationship between the factors explored and other adverse environmental risks which often co-occur and may be underlying these relationships.

The prospective nature of the parent study, including measurement of a broad range of risk factors and robust measurement of developmental outcomes in an LMIC

setting, is a strength of this study. However, there are several limitations. The current study used cross sectional data, therefore we cannot assert causality. Maternal sexual IPV prevalence was very low in our sample (4%) and this likely affected power to discern potential associations. We also did not investigate longitudinal exposures or cumulative impact of maternal depression or alcohol use; these were only investigated as recent exposures. Further, although the BSID-III is a well recognised tool, further standardisation in sub-Saharan African settings is required. For example, the categorisation of delay is based on scaled scores using normative US data that might not be generalisable to a South African population. However, our data from the parent study has shown that raw scores have similar patterns to the results based on delay categorisation. There were some differences in characteristics of those included versus excluded in the present study, namely HIV exposure and maternal employment. Maternal employment was lower in the included sub-sample (41% vs 51%), however, household income was similar across groups. Furthermore, maternal employment was not associated with any outcomes investigated in bivariate analyses and adjustment for this did not alter any of the key associations. HIV exposure was higher in the included sub-sample (24% versus 18%), however, this would likely bolster potential associations. In addition, approximately one-third of children who were still active in the study at two years were not included in the analysis due to incomplete data. However, results were similar when associations were explored in the imputed dataset.

Conclusions

In summary, our results highlight the need for programs that aim to reduce IPV as well as early life prevention efforts to mitigate the impact of maternal IPV on children. Maternal IPV was a consistent predictor of poor child developmental

For peer review only - http://bmjopen.bmj.com/site/about/guidelines.xhtml

outcomes; furthermore, emotional IPV emerged as a uniquely deleterious risk factor for child developmental outcomes. Further work is needed to understand the mechanisms underlying this relationship, exploring maternal depression and alcohol use in other samples as well as additional potential mechamisms. Given our findings, it is important for public health professionals and policy makers to ensure that maternal emotional IPV is included in training programs for health care staff as well as in screening and referral efforts. Lastly, more research investigating emotional or psychological IPV and adverse health outcomes is needed. Critically, in our setting, the majority of pregnant and early postpartum women access healthcare, providing an important, potentially under-utilized opportunity to intervene early to attempt to shield children from the negative effects of IPV.

Data sharing

Collaborations for the analysis of data are welcome; the parent study has a large and active group of investigators and postgraduate students and many have successfully partnered with students or researchers from other institutions. Researchers who are interested in datasets or collaborations can find more information on our website [http://www.paediatrics.uct.ac.za/scah/dclhs].

Competing interests

The authors have no conflicts of interest to declare.

Author contributions

WB, KD and SH conceptualized the study aims and methodology. WB with guidance from RM, conducted all analyses. HJZ is principal investigator of the parent study; DS leads the psychosocial study aspects; CW and KD are coinvestigators and were

For peer review only

responsible for implementation, training and quality control of developmental assessments. All authors provided intellectual input and reviewed and approved the final manuscript.

References

1. Garcia-Moreno C, Jansen HA, Ellsberg M, Heise L, Watts CH. Prevalence of intimate partner violence: findings from the WHO multi-country study on women's health and domestic violence. *Lancet* 2006; **368**(9543): 1260-9.
2. Dunkle KL, Jewkes RK, Brown HC, et al. Prevalence and patterns of gender-based violence and revictimization among women attending antenatal clinics in Soweto, South Africa. *Am J Epidemiol* 2004; **160**(3): 230-9.
3. Jewkes R, Penn-Kekana L, Levin J, Ratsaka M, Schrieber M. Prevalence of emotional, physical and sexual abuse of women in three South African provinces. *S Afr Med J* 2001; **91**(5): 421-8.
4. Osofsky JD. Prevalence of children's exposure to domestic violence and child maltreatment: implications for prevention and intervention. *Clin Child Fam Psychol Rev* 2003; **6**(3): 161-70.
5. Pinheiro, P. S. *World Report on Violence Against Children* (United Nations, Geneva, 2006). Retrieved 28 Apr 2020, from https://www.unicef.org/violencestudy/reports/SG_violencestudy_en.pdf.
6. Kernic MA, Holt VL, Wolf ME, McKnight B, Huebner CE, Rivara FP. Academic and School Health Issues Among Children Exposed to Maternal Intimate Partner Abuse. *Arch Pediatr Adolesc Med* 2002; **156**(6): 549-55.
7. Vu NL, Jouriles EN, McDonald R, Rosenfield D. Children's exposure to intimate partner violence: A meta-analysis of longitudinal associations with child adjustment problems. *Clin Psychol Rev* 2016; **46**: 25-33.
8. Wolfe DA, Crooks CV, Lee V, McIntyre-Smith A, Jaffe PG. The effects of children's exposure to domestic violence: a meta-analysis and critique. *Clin Child Fam Psychol Rev* 2003; **6**(3): 171-87.
9. Bair-Merritt MH, Blackstone M, Feudtner C. Physical health outcomes of childhood exposure to intimate partner violence: a systematic review. *Pediatrics* 2006; **117**(2): e278-90.
10. Gale CR, Cooper R, Craig L, et al. Cognitive function in childhood and lifetime cognitive change in relation to mental wellbeing in four cohorts of older people. *PLoS One* 2012; **7**(9): e44860.
11. Howell KH, Barnes SE, Miller LE, Graham-Bermann SA. Developmental variations in the impact of intimate partner violence exposure during childhood. *J Inj Violence Res* 2016; **8**(1): 43-57.
12. Udo IE, Sharps P, Bronner Y, Hossain MB. Maternal Intimate Partner Violence: Relationships with Language and Neurological Development of Infants and Toddlers. *Matern Child Health J* 2016; **20**(7): 1424-31.
13. Neamah HH, Sudfeld C, McCoy DC, et al. Intimate Partner Violence, Depression, and Child Growth and Development. *Pediatrics* 2018; **142**(1).
14. Shonkoff JP, Garner AS. The lifelong effects of early childhood adversity and toxic stress. *Pediatrics* 2012; **129**(1): e232-46.

For peer review only

15. Enlow MB, Egeland B, Blood EA, Wright RO, Wright RJ. Interpersonal trauma exposure and cognitive development in children to age 8 years: a longitudinal study. *J Epidemiol Community Health* 2012; **66**(11): 1005-10.
16. Kramer A, Lorenzon D, Mueller G. Prevalence of intimate partner violence and health implications for women using emergency departments and primary care clinics. *Womens Health Issues* 2004; **14**(1): 19-29.
17. Yoshihama M, Horrocks J, Kamano S. The role of emotional abuse in intimate partner violence and health among women in Yokohama, Japan. *Am J Public Health* 2009; **99**(4): 647-53.
18. Coker AL, Davis KE, Arias I, et al. Physical and mental health effects of intimate partner violence for men and women. *Am J Prev Med* 2002; **23**(4): 260-8.
19. Okafor CN, Barnett W, Zar HJ, et al. Associations of Emotional, Physical, or Sexual Intimate Partner Violence and Depression Symptoms Among South African Women in a Prospective Cohort Study. *J Interpers Violence* 2018: 886260518796522.
20. Seedat M, Van Niekerk A, Jewkes R, Suffla S, Ratele K. Violence and injuries in South Africa: prioritising an agenda for prevention. *Lancet* 2009; **374**(9694): 1011-22.
21. Coovadia H, Jewkes R, Barron P, Sanders D, McIntyre D. The health and health system of South Africa: historical roots of current public health challenges. *Lancet* 2009; **374**(9692): 817-34.
22. Zembe YZ, Townsend L, Thorson A, Silberschmidt M, Ekstrom AM. Intimate Partner Violence, Relationship Power Inequity and the Role of Sexual and Social Risk Factors in the Production of Violence among Young Women Who Have Multiple Sexual Partners in a Peri-Urban Setting in South Africa. *PLoS One* 2015; **10**(11): e0139430.
23. Atkinson L, Paglia A, Coolbear J, Niccols A, Parker KC, Guger S. Attachment security: a meta-analysis of maternal mental health correlates. *Clin Psychol Rev* 2000; **20**(8): 1019-40.
24. Myers B. LJ, Fakier N. Access to substance abuse treatment in the Cape Town Metropole: Final report. *South African Medical Research Council* 2007.
25. Jester JM, Jacobson SW, Sokol RJ, Tuttle BS, Jacobson JL. The influence of maternal drinking and drug use on the quality of the home environment of school-aged children. *Alcohol Clin Exp Res* 2000; **24**(8): 1187-97.
26. Jansson LM. Maternal Alcohol Use During Lactation and Child Development. *Pediatrics* 2018; **142**(2).
27. Rochat TJ, Houle B, Stein A, Mitchell J, Bland RM. Maternal alcohol use and children's emotional and cognitive outcomes in rural South Africa. *S Afr Med J* 2019; **109**(7): 526-34.
28. Barnes J, Theule J. Maternal depression and infant attachment security: A meta-analysis. *Infant Ment Health J* 2019; **40**(6): 817-34.
29. National Research Council (US) and Institute of Medicine (US) Committee on Depression, Parenting Practices, and the Healthy Development of Children. *Depression in Parents, Parenting, and Children: Opportunities to Improve Identification, Treatment, and Prevention.* 2009. Retrieved 10 Jul 2020, from: https://www.ncbi.nlm.nih.gov/books/NBK215128/.
30. Stein A, Malmberg LE, Sylva K, Barnes J, Leach P. The influence of maternal depression, caregiving, and socioeconomic status in the post-natal year on children's language development. *Child Care Health Dev* 2008; **34**(5): 603-12.

For peer review only

31. Kitzmann KM, Gaylord NK, Holt AR, Kenny ED. Child witnesses to domestic violence: a meta-analytic review. *J Consult Clin Psychol* 2003; **71**(2): 339-52.
32. Stein DJ, Koen N, Donald KA, et al. Investigating the psychosocial determinants of child health in Africa: The Drakenstein Child Health Study. *J Neurosci Methods* 2015.
33. Zar HJ, Barnett W, Myer L, Stein DJ, Nicol MP. Investigating the early-life determinants of illness in Africa: the Drakenstein Child Health Study. *Thorax* 2015; **70**(6): 592-4.
34. Donald KA, Hoogenhout M, du Plooy CP, et al. Drakenstein Child Health Study (DCHS): investigating determinants of early child development and cognition. *BMJ Paediatr Open* 2018; **2**(1): e000282.
35. Myer L, Stein DJ, Grimsrud A, Seedat S, Williams DR. Social determinants of psychological distress in a nationally-representative sample of South African adults. *Soc Sci Med* 2008; **66**(8): 1828-40.
36. Pellowski J, Wedderburn C, Stadler JAM, et al. Implementation of prevention of mother-to-child transmission (PMTCT) in South Africa: outcomes from a population-based birth cohort study in Paarl, Western Cape. *BMJ Open* 2019; **9**(12): e033259.
37. Budree S, Stein DJ, Brittain K, et al. Maternal and infant factors had a significant impact on birthweight and longitudinal growth in a South African birth cohort. *Acta Paediatr* 2017; **106**(11): 1793-801.
38. Jewkes R. Intimate partner violence: causes and prevention. *Lancet* 2002; **359**(9315): 1423-9.
39. Shamu S, Abrahams N, Zarowsky C, Shefer T, Temmerman M. Intimate partner violence during pregnancy in Zimbabwe: a cross-sectional study of prevalence, predictors and associations with HIV. *Trop Med Int Health* 2013; **18**(6): 696-711.
40. Dunkle KL, Jewkes RK, Brown HC, Gray GE, McIntryre JA, Harlow SD. Gender-based violence, relationship power, and risk of HIV infection in women attending antenatal clinics in South Africa. *Lancet* 2004.
41. Beck AT, Steer RA, Carbin MG. Psychometric properties of the Beck Depression Inventory: Twenty-five years of evaluation. *Clin Psychol Rev* 1988; **8**(1): 77-100.
42. Kagee A, Nel A, Saal W. Factor structure of the Beck Depression Inventory-II among South Africans receiving antiretroviral therapy. *AIDS Care* 2014; **26**(2): 257-62.
43. Lasa L, Ayuso-Mateos JL, Vázquez-Barquero JL, Díez-Manrique FJ, Dowrick CF. The use of the Beck Depression Inventory to screen for depression in the general population: a preliminary analysis. *J Affect Disord* 2000; **57**(1-3): 261-5.
44. Humeniuk R, Ali R, Babor TF, et al. Validation of the Alcohol, Smoking And Substance Involvement Screening Test (ASSIST). *Addiction* 2008; **103**(6): 1039-47.
45. World Health Organization. 2010. *The Alcohol, Smoking and Substance Involvement Screening Test (ASSIST) Manual for use in primary care.* Retrieved 28 June 2020, from http://www.who.int/substance_abuse/publications/assist/en/.
46. Bayley N. Pearson; Bloomington, MN: 2006. Bayley scales of infant and toddler development, 3rd edition-technical manual.

For peer review only

47. Rademeyer V, Jacklin L. A study to evaluate the performance of black South African urban infants on the Bayley Scales of Infant Development III. *SA J Child Health* 2013; **7**: 54-9.
48. Barnett W, Brittain K, Sorsdahl K, Zar HJ, Stein DJ. Maternal participant experience in a South African birth cohort study enrolling healthy pregnant women and their infants. *Philos Ethics Humanit Med* 2016; **11**(1): 3.
49. Villar J, Papageorghiou AT, Pang R, et al. The likeness of fetal growth and newborn size across non-isolated populations in the INTERGROWTH-21st Project: the Fetal Growth Longitudinal Study and Newborn Cross-Sectional Study. *Lancet Diabetes Endocrinol* 2014; **2**(10): 781-92.
50. World Health Organization. 2006. *'WHO Child Growth Standards: length/height-for-age, weight-for-age, weight-for-length, weight-for-height and body mass index-for-age: methods and development.* Geneva: World Health Organization. Acta Paediatr Suppl, 2006; 450: 76–85.
51. Jeong J, Adhia A, Bhatia A, McCoy DC, Yousafzai AK. Intimate Partner Violence, Maternal and Paternal Parenting, and Early Child Development. *Pediatrics* 2020; **145**(6): e20192955.
52. Rodriguez VJ, Peltzer K, Matseke G, Weiss SM, Shine A, Jones DL. Pre- and postnatal exposure to intimate partner violence among South African HIV-infected mothers and infant developmental functioning at 12 months of age. *Arch Womens Ment Health* 2018; **21**(6): 707-13.
53. Tiwari A, Chan K, Fong D, et al. The impact of psychological abuse by an intimate partner on the mental health of pregnant women. *Bjog* 2008; **115**(3): 377-84.
54. Myers B, Koen N, Donald KA, et al. Effect of Hazardous Alcohol Use During Pregnancy on Growth Outcomes at Birth: Findings from a South African Cohort Study. *Alcohol Clin Exp Res* 2018; **42**(2): 369-77.
55. Bergin C, McCollough P. Attachment in substance-exposed toddlers: The role of caregiving and exposure. *Infant Ment Health J* 2009; **30**(4): 407-23.
56. Eiden RD, Edwards EP, Leonard KE. Mother-infant and father-infant attachment among alcoholic families. *Dev Psychopathol* 2002; **14**(2): 253-78.
57. Galler JR, Harrison RH, Ramsey F, Forde V, Butler SC. Maternal depressive symptoms affect infant cognitive development in Barbados. *J Child Psychol Psychiatry* 2000; **41**(6): 747-57.
58. Morais MdLSe, Lucci TK, Otta E. Postpartum depression and child development in first year of life. *Estudos de Psicologia (Campinas)* 2013; **30**: 7-17.

For peer review only

Table 1. Sample maternal and child psychosocial, demographic and clinical characteristics, n=626*

	Total *n=626*
Intimate Partner Violence (24 months)	
Emotional score, median (IQR)	0 (0, 0)
Emotional (above threshold)	102 (16)
Physical score, median (IQR)	0 (0, 0)
Physical (above threshold)	107 (17)
Sexual score, median (IQR)	0 (0, 0)
Sexual (above threshold)	25 (4)
Any sub-type score, median (IQR)	0 (0,0)
Any sub-type (above threshold)	138 (22)
Sociodemographics (24 months)	
Maternal education (secondary not completed)	233 (37)
Mother employed	225 (41)
Mother married/partnered	268 (49)
Household Income	
<R5,000 (approximately 300 USD)	445 (73)
>R5,000	164 (27)
Recruitment site: TC Newman	274 (440
Physical variables	
Birth weight z-score, median (IQR)**	-0.59 (-1.34, 0.16)
Child age at assessment (months), median (IQR)	24.05 (23.79, 24.31)
HIV exposed**	153 (24)
Length for age z-score at 24 months, median (IQR)	-1.16 (-1.89, -0.52)
Weight for age z-score at 24 months, median (IQR)	-0.31 (-1.10, 0.43)
Weight for length z-score at 24 months, median (IQR)	0.28 (-0.48, 1.21)
Psychosocial variables (24 months)	
Depression score, median (IQR)	0 (0, 3)
Depression (above threshold)	64 (11)
Alcohol dependence score, median (IQR)	0 (0, 0)
Alcohol dependence (categories)	
Low/No dependence, n (%)	160 (27)
Moderate dependence, n (%)	33 (6)
High dependence, n (%)	22 (4)

Notes: Median (IQR ranges reported for anthropometry data, intimate partner violence scores as well as depression and alcohol scores. Frequencies (percentages) reported for all other variables. Intimate partner violence, above threshold exposure defined as more than an isolated incident within each sub-type. Depression threshold defined as EPDS score of >=13. Alcohol dependence categories defined as: low/no risk (0-10), moderate risk (11-26) and high risk (>26), with the likelihood of alcohol dependence.
*Includes data from mothers or children included in complete case dataset.
**Birthweight and HIV exposure collected at birth; all other variables collected at 2 years of child age.

For peer review only

Table 2. Developmental outcomes of children at 2 years of age by child sex

	Total	Female	Male	p-value
Sample	*n=625*	*n=303*	*n=322*	
Cognitive scores, mean (SD)	85.74 (8.84)	86.44 (8.56)	85.08 (9.06)	0.035
Cognitive categories				
No delay, n (%)	425 (68)	218 (72)	207 (64)	
Suboptimal development, n (%)	173 (28)	75 (25)	98 (30)	
Delayed, n (%)	28 (4)	10 (3)	18 (6)	0.082
Sample	*n=598*	*n=289*	*n=309*	
Language scores, mean (SD)	84.45 (11.39)	86.57 (11.35)	82.47 (11.08)	<0.001
Language categories				
No delay, n (%)	325 (52)	178 (59)	147 (46)	
Suboptimal development, n (%)	253 (40)	110 (36)	143 (44)	
Delayed, n (%)	48 (8)	15 (5)	33 (10)	0.001
Sample	*n=592*	*n=289*	*n=303*	
Motor scores, mean (SD)	93.62 (11.89)	94.40 (12.12)	92.86 (11.63)	0.111
Motor categories				
No delay, n (%)	524 (84)	257 (85)	267 (83)	
Suboptimal development, n (%)	95 (15)	41 (14)	54 (17)	
Delayed, n (%)	7 (1)	5 (2)	2 (1)	0.270

Continuous composite scores are presented as mean (SD). Categorical data presented as frequencies (percentages). Categories were defined as 'delay' (<2 standard deviations below mean), 'suboptimal development' (<1 standard deviation below mean) and 'no delay'. P-values presented are comparing sex differences.

For peer review only

Table 3. Univariate associations between intimate partner violence, covariates and proposed mediators and composite scores for developmental domains in complete case analysis.

	**Composite Cognitive (n=549)**	**Composite Language (n=598)**	**Composite Motor (n=521)**
	Unadjusted<br>Coefficient (95% CI)	Unadjusted<br>Coefficient (95% CI)	Unadjusted<br>Coefficient (95% CI)
Intimate Partner Violence (IPV)			
Emotional IPV score	-0.32 (-0.58, -0.06)*	-0.35 (-0.69, -0.01)*	-0.58 (-0.93, -0.24)*
Physical IPV score	-0.18 (-0.42, 0.05)	-0.23 (-0.54, 0.08)	-0.42 (-0.73, -0.11)*
Sexual IPV score	-0.15 (-0.97, 0.67)	-0.16 (-1.29, 0.97)	-0.49 (-1.63, 0.65)
Sociodemographics			
Maternal education (not completed secondary)	-1.31 (-2.70, 0.08)	-2.86 (-4.61, -1.11)*	-0.93 (-2.78, 0.92)
Mother employed	0.60 (-0.87, 2.08)	0.34 (-1.57, 2.27)	0.68 (-1.29, 2.65)
Mother married/partnered	-0.52 (-1.98, 0.93)	0.47 (-1.43, 2.37)	0.01 (-1.94, 1.95)
Household Income			
>R5000	0.73 (-0.92, 2.38)	0.32 (-1.80, 2.45)	1.63 (-0.55, 3.82)
Recruitment site: TC Newman	1.19 (-0.16, 2.53)	3.05 (1.35, 4.76)**	1.13 (-0.66, 2.93)
Physical variables			
Birth weight z-score	0.42 (-0.17, 1.01)	0.84 (0.09, 1.58)*	0.62 (-0.17, 1.39)
Male	-1.66 (-3.01, -0.32)*	-4.27 (-5.96, -2.59)**	-1.52 (-3.31, 0.27)
Child age (months)	-0.94 (-2.23, 0.34)	-0.10 (-1.74, 1.53)	-2.81 (-4.49, -1.12)*
HIV exposed	-1.71 (-3.30, -0.11)*	-3.90 (-5.93, -1.87)**	-0.76 (-2.89, 1.38)
Length for age z-score	0.97 (0.36, 1.58)*	0.77 (-0.01, 1.55)	0.13 (-0.70, 0.97)
Weight for age z-score	0.92 (0.32, 1.51)*	0.64 (-0.12, 1.40)	0.68 (-0.19, 1.47)
Weight for length z-score	0.61 (0.02, 1.19)*	-0.34 (-0.41, 1.09)	0.84 (0.05, 1.63)*
Proposed Psychosocial mediators			
Depression score	-0.02 (-0.11, 0.08)	-0.06 (-0.18, 0.06)	-0.12 (-0.25, 0.01)
Alcohol dependence score	-0.07 (-0.17, 0.03)	-0.03 (-0.16, 0.11)	0.01 (-0.13, 0.15)

Notes: All variables measured at 2 years of child age except birth weight, sex and HIV-exposure which were captured at birth. IPV sub-types, depression and alcohol dependence were included as continuous variables in all models.
Abbreviations: IPV=intimate partner violence.
*p<0.05; **p<0.001;

For peer review only

Table 4. Multivariable linear regression demonstrating the adjusted association of intimate partner violence and composite scores for developmental domains in complete case analysis sample.

	**Composite Cognitive (n=549)**	**Composite Language (n=598)**	**Composite Motor (n=521)**	
	Adjusted# Coefficient (95% CI)	Adjusted^ Coefficient (95% CI)	Adjusted+ Coefficient (95% CI)	Adjusted+ Coefficient (95% CI)
Emotional IPV score	-0.32 (-0.60, -0.04)*	-0.35 (-0.69, -0.01)*	-0.58 (-0.95, -0.20)*	
Physical IPV score				-0.42 (-0.75, -0.09)*

Adjusted models were run only where IPV sub-type was associated with outcome explored (p<0.05) in bivariate analyses. For cognitive and language scores, only emotional IPV was associated in bivariate analyses and therefore run in multivariable models. Due to collinearity, emotional and physical IPV were run in separate multivariable models for motor development. IPV sub-types, depression and alcohol dependence were included as continuous variables in all models.
Abbreviations: IPV=intimate partner violence.
*p<0.05; **p<0.001
#Adjusted for child sex, HIV exposure, length-for-age z-scores, weight-for-age z-scores and weight-for-length z-scores at 24 months.
^Adjusted for maternal education, recruitment site, weight-for-age z-score at birth, child sex and HIV exposure.
+Adjusted for child age at assessment and weight-for-length z-scores at 24 months.

For peer review only

Supplemental Table 1. Comparison of psychosocial, demographic and clinical data between children included in complete case developmental models and those who are not.

	In Development models	Not in Development model	p-value
Intimate Partner Violence (IPV)			
Emotional IPV score, median (IQR)	0 (0, 0)	0 (0, 0)	0.669
Physical IPV score, median (IQR)	0 (0, 0)	0 (0, 0)	0.213
Sexual IPV score, median (IQR)	0 (0, 0)	0 (0, 0)	0.160.
Sociodemographics			
Maternal education (did not complete secondary)	393 (63)	302 (58)	0.132
Mother employed	225 (41)	99 (51)	0.022*
Mother married/partnered	268 (49)	99 (51)	0.718
Household Income			
<R5000	445 (73)	122 (63)	0.671
>R5000	164 (27)	47 (24)	
Recruitment site: TC Newman	274 (44)	235 (45)	0.586
Physical variables			
Birth weight z-score, median (IQR)	-0.59 (-1.34, 0.16)	-0.51 (-1.22, -0.00)	0.744
Male sex	321 (51)	269 (52)	0.774
HIV exposed	153 (24)	94 (18)	0.010*
Length for age z-scores, median (IQR)	-1.10 (-1.80, -0.34)	-1.03 (-1.85, -0.28)	0.846
Weight for age z-scores, median (IQR)	-0.31 (-1.10, 0.43)	-0.24 (-1.01, 0.40)	0.6682
Weight for length z-scores, median (IQR)	0.28 (-0.48, 1.13)	0.43 (-0.40, 1.09)	0.7542
Psychosocial factors			
Depression score, median (IQR)	0 (0, 3)	0 (0, 5)	0.415
Alcohol dependence score, median (IQR)	0 (0,0)	0 (0,0)	0.068

Data are presented as n(%) or median IQR). Birthweight and HIV exposure collected at birth; all other variables collected at 2 years of child age. Abbreviation: IPV=intimate partner violence.
*p<0.05; **p<0.001

For peer review only

Supplemental Table 2. Univariate associations between intimate partner violence, covariates and proposed mediators and composite scores for developmental domains in the multiple-imputation analysis sample.

	Composite Cognitive	Composite Language	Composite Motor
	Unadjusted Coefficient (95% CI)	Unadjusted Coefficient (95% CI)	Unadjusted Coefficient (95% CI)
Intimate Partner Violence (IPV)			
Emotional IPV score	-0.33 (-0.60, -0.06)*	-0.34 (-0.68, -0.01)*	-0.51 (-0.86, -0.16)*
Physical IPV score	-0.19 (-0.43, 0.05)	-0.20 (-0.50, 0.11)	-0.39 (-0.70, -0.09)*
Sexual IPV score	-0.13 (-0.98, 0.71)	-0.16 (-1.36, 1.04)	-0.41 (-1.58, 0.75)
Sociodemographics			
Maternal education (not completed secondary)	-1.31 (-2.69, 0.07)	-3.20 (-4.94, -1.45)**	-1.45 (-3.30, 0.40)
Mother employed	0.48 (-1.02, 1.99)	0.13 (-1.81, 2.07)	0.41 (-1.55, 2.38)
Mother married/partnered	-0.59 (-2.07, 0.89)	0.26 (-1.64, 2.16)	0.15 (-1.88, 2.18)
Household Income			
>R5000	0.73 (-0.92, 2.38)	1.00 (-1.14, 3.13)	1.96 (-0.28, 4.20)
TC Newman	1.20 (-0.15, 2.54)	3.12 (1.41, 4.84)**	0.89 (-0.91, 2.68)
Physical variables			
Birth weight z-score	0.44 (-0.15, 1.03)	0.68 (-0.07, 1.42)	0.58 (-0.21, 1.36)
Male	-1.67 (-3.01, -0.33)*	-4.07 (-5.77, -2.37)**	-1.70 (-3.49, 0.09)
Child age (months)	-0.95 (-2.24, 0.33)	-0.12 (-1.77, 1.53)	-2.81 (-4.50, -1.11)*
HIV exposed	-1.70 (-3.29, -0.10)*	-3.77 (-5.82, -1.72)**	-0.59 (-2.72, 1.54)
Length for age z-score	0.92 (0.33, 1.52)*	0.67 (-0.09, 1.42)	0.26 (-0.54, 1.06)
Weight for age z-score	0.84 (0.26, 1.43)*	0.64 (-0.10, 1.37)	0.82 (0.04, 1.60)*
Weight for length z-score	0.50 (-0.08, 1.08)	0.39 (-0.34, 1.11)	0.88 (0.10, 1.66)*
Proposed Psychosocial mediators			
Depression score	-0.02 (-0.12, 0.08)	-0.06 (-0.18, 0.07)	-0.11 (-0.24, 0.02)
Alcohol dependence score	-0.08 (-0.19, 0.03)	-0.03 (-0.17, 0.11)	0.03 (-0.11, 0.16)

Birthweight and HIV exposure collected at birth; all other variables collected at 2 years of child age. IPV sub-types, depression and alcohol dependence were included as continuous variables in all models.
Abbreviation: IPV= intimate partner violence.
*p<0.05, **p<0.001

Supplemental Table 3. Multivariable linear regression demonstrating the association of intimate partner violence with composite scores for developmental domains in the multiple-imputation analysis sample.

	Cognitive scores	Language scores	Motor scores	
	Adjusted# Coefficient (95% CI)	Adjusted^ Coefficient (95% CI)	Adjusted+ Coefficient (95% CI)	Adjusted+ Coefficient (95% CI)
Emotional IPV score	-0.30 (-0.57, -0.03)*	-0.35 (-0.68, -0.01)	-0.52 (-0.87, -0.17)*	
Physical IPV score				-0.39 (-0.70, -0.09)*

Adjusted models were run only where IPV sub-type was associated (p<0.05) with outcome explored in bivariate analyses. For cognitive and language scores, only emotional IPV was associated in bivariate analyses and therefore run in multivariable models. Due to collinearity, emotional and physical IPV were run in separate multivariable models for motor development. Adjusted models were run including same covariates as the complete case analysis, as noted below. IPV sub-types, depression and alcohol dependence were included as continuous variables in all models.
Abbreviations: IPV=intimate partner violence.
*p<0.05; **p<0.001
#Adjusted for child sex, HIV exposure, length-for-age z-scores, weight-for-age z-scores and weight-for-length z-scores at 24 months.
^Adjusted for maternal education, recruitment site, weight-for-age z-score at birth, child sex and HIV exposure.
+Adjusted for child age at assessment and weight-for-length z-scores at 24 months.

For peer review only

Supplemental Table 4. Univariate associations between proposed mediators and intimate partner violence in complete case sample.

	**Emotional IPV** Coefficient (95% CI)	**Physical IPV** Coefficient (95% CI)
Depression score	1.01 (0.80, 1.22)**	0.68 (0.49, 0.87)**
Alcohol dependence score	0.34 (0.13, 0.56)*	0.30 (0.12, 0.49)*

IPV sub-types, depression and alcohol dependence were included as continuous variables in all models.
Abbreviation: IPV = intimate partner violence.
*p<0.05, **p<0.001

Supplemental Table 5. Univariate and adjusted associations between proposed mediators and developmental outcomes in complete case sample.

		**Cognitive scores#** Coefficient (95% CI)	**Language scores^** Coefficient (95% CI)	**Motor scores+** Coefficient (95% CI)
Univariate	Depression	-0.02 (-0.11, 0.08)	-0.06 (-0.18, 0.06)	-0.12 (-0.25, 0.01)
	Alcohol	-0.07 (-0.17, 0.03)	-0.03 (-0.16, 0.11)	0.01 (-0.13, 0.15)
Adjusted	Depression	0.02 (-0.08, 0.13)	-0.05 (-0.18, 0.07)	-0.04 (-0.19, 0.10)
	Alcohol	-0.06 (-0.17, 0.05)	0.01 (-0.12, 0.14)	0.05 (-0.10, 0.20)

Notes: Adjusted associations between proposed mediators and developmental domains were run using linear regression. Depression and alcohol were run separately in adjusted regression models. IPV sub-types, depression and alcohol dependence were included as continuous variables in all models.
Abbreviation: IPV = intimate partner violence.
#Adjusted for intimate partner violence, child sex, HIV exposure, length-for-age z-scores, weight-for-age z-scores and weight-for-length z-scores at 24 months.
^Adjusted for intimate partner violence, maternal education, recruitment site, weight-for-age z-score at birth, child sex and HIV exposure.
+Adjusted for intimate partner violence, child age at assessment and weight-for-length z-scores at 24 months.

For peer review only

STROBE Statement—Checklist of items that should be included in reports of ***cohort studies***

	Item No	Recommendation	Page No
**Title and abstract**	1	(*a*) Indicate the study's design with a commonly used term in the title or the abstract<br>(*b*) Provide in the abstract an informative and balanced summary of what was done and what was found	1<br>2
**Introduction**			
Background/rationale	2	Explain the scientific background and rationale for the investigation being reported	3-5
Objectives	3	State specific objectives, including any prespecified hypotheses	5
**Methods**			
Study design	4	Present key elements of study design early in the paper	5
Setting	5	Describe the setting, locations, and relevant dates, including periods of recruitment, exposure, follow-up, and data collection	5/6
Participants	6	(*a*) Give the eligibility criteria, and the sources and methods of selection of participants. Describe methods of follow-up<br>(*b*) For matched studies, give matching criteria and number of exposed and unexposed	6<br>NA
Variables	7	Clearly define all outcomes, exposures, predictors, potential confounders, and effect modifiers. Give diagnostic criteria, if applicable	6-9
Data sources/ measurement	8*	For each variable of interest, give sources of data and details of methods of assessment (measurement). Describe comparability of assessment methods if there is more than one group	6-9
Bias	9	Describe any efforts to address potential sources of bias	11
Study size	10	Explain how the study size was arrived at	11
Quantitative variables	11	Explain how quantitative variables were handled in the analyses. If applicable, describe which groupings were chosen and why	10-11
Statistical methods	12	(*a*) Describe all statistical methods, including those used to control for confounding<br>(*b*) Describe any methods used to examine subgroups and interactions<br>(*c*) Explain how missing data were addressed<br>(*d*) If applicable, explain how loss to follow-up was addressed<br>(*e*) Describe any sensitivity analyses	10-11<br>N/A<br>Supplemental<br><br>Supplemental
**Results**			
Participants	13*	(a) Report numbers of individuals at each stage of study—eg numbers potentially eligible, examined for eligibility, confirmed eligible, included in the study, completing follow-up, and analysed<br>(b) Give reasons for non-participation at each stage<br>(c) Consider use of a flow diagram	11<br><br>11
Descriptive data	14*	(a) Give characteristics of study participants (eg demographic, clinical, social) and information on exposures and potential confounders<br>(b) Indicate number of participants with missing data for each variable of interest<br>(c) Summarise follow-up time (eg, average and total amount)	12<br>Table 1
Outcome data	15*	Report numbers of outcome events or summary measures over time	12, Table 2

For peer review only - http://bmjopen.bmj.com/site/about/guidelines.xhtml

Main results	16	(*a*) Give unadjusted estimates and, if applicable, confounder-adjusted estimates and their precision (eg, 95% confidence interval). Make clear which confounders were adjusted for and why they were included<br>(*b*) Report category boundaries when continuous variables were categorized<br>(*c*) If relevant, consider translating estimates of relative risk into absolute risk for a meaningful time period	12-13 Tables 3,4<br>7-8 Tables 1,2
Other analyses	17	Report other analyses done—eg analyses of subgroups and interactions, and sensitivity analyses	Supplemental tables
**Discussion**			
Key results	18	Summarise key results with reference to study objectives	13-14
Limitations	19	Discuss limitations of the study, taking into account sources of potential bias or imprecision. Discuss both direction and magnitude of any potential bias	16-17
Interpretation	20	Give a cautious overall interpretation of results considering objectives, limitations, multiplicity of analyses, results from similar studies, and other relevant evidence	17
Generalisability	21	Discuss the generalisability (external validity) of the study results	16-17
**Other information**			
Funding	22	Give the source of funding and the role of the funders for the present study and, if applicable, for the original study on which the present article is based	1

*Give information separately for exposed and unexposed groups.

Note: An Explanation and Elaboration article discusses each checklist item and gives methodological background and published examples of transparent reporting. The STROBE checklist is best used in conjunction with this article (freely available on the Web sites of PLoS Medicine at http://www.plosmedicine.org/, Annals of Internal Medicine at http://www.annals.org/, and Epidemiology at http://www.epidem.com/). Information on the STROBE Initiative is available at http://www.strobe-statement.org.

For peer review only

BMJ Open

Maternal emotional and physical intimate partner violence and early child development: investigating mediators in a cross sectional study in a South African birth cohort.

Journal:	*BMJ Open*
Manuscript ID	bmjopen-2020-046829.R1
Article Type:	Original research
Date Submitted by the Author:	12-May-2021
Complete List of Authors:	Barnett, Whitney; University of Cape Town Faculty of Health Sciences, Department of Paediatrics and Child Health & South African Medical Research Council Unit on Child & Adolescent Health<br>Halligan, Sarah; University of Bath, Department of Psychology; University of Cape Town Faculty of Health Sciences, Department of Psychiatry and Mental Health & South African Medical Research Council Unit on Risk and Resilience in Mental Disorders<br>Wedderburn, Catherine; London School of Hygiene & Tropical Medicine, Department of Clinical Research; University of Cape Town, Department of Paediatrics and Child Health & South African Medical Research Council Unit on Child & Adolescent Health<br>MacGinty, Rae; University of Cape Town Faculty of Health Sciences, Department of Paediatrics and Child Health & South African Medical Research Council Unit on Child & Adolescent Health<br>Hoffman, Nadia; University of Cape Town Faculty of Health Sciences, Department of Psychiatry and Mental Health & South African Medical Research Council Unit on Risk and Resilience in Mental Disorders<br>Zar, Heather; University of Cape Town Faculty of Health Sciences, Department of Paediatrics and Child Health & South African Medical Research Council Unit on Child & Adolescent Health<br>Stein, Dan; University of Cape Town Faculty of Health Sciences, Department of Psychiatry and Mental Health & South African Medical Research Council Unit on Risk and Resilience in Mental Disorders; University of Cape Town Faculty of Health Sciences, Neuroscience Institute<br>Donald, Kirsten; University of Cape Town Faculty of Health Sciences, Division of Developmental Paediatrics, Department of Paediatrics and Child Health; University of Cape Town Faculty of Health Sciences, Neuroscience Institute
**Primary Subject Heading**:	Paediatrics
Secondary Subject Heading:	Global health, Public health
Keywords:	Developmental neurology & neurodisability < PAEDIATRICS, PUBLIC HEALTH, TRAUMA MANAGEMENT

I, the Submitting Author has the right to grant and does grant on behalf of all authors of the Work (as defined in the below author licence), an exclusive licence and/or a non-exclusive licence for contributions from authors who are: i) UK Crown employees; ii) where BMJ has agreed a CC-BY licence shall apply, and/or iii) in accordance with the terms applicable for US Federal Government officers or employees acting as part of their official duties; on a worldwide, perpetual, irrevocable, royalty-free basis to BMJ Publishing Group Ltd ("BMJ") its licensees and where the relevant Journal is co-owned by BMJ to the co-owners of the Journal, to publish the Work in this journal and any other BMJ products and to exploit all rights, as set out in our licence.

The Submitting Author accepts and understands that any supply made under these terms is made by BMJ to the Submitting Author unless you are acting as an employee on behalf of your employer or a postgraduate student of an affiliated institution which is paying any applicable article publishing charge ("APC") for Open Access articles. Where the Submitting Author wishes to make the Work available on an Open Access basis (and intends to pay the relevant APC), the terms of reuse of such Open Access shall be governed by a Creative Commons licence – details of these licences and which Creative Commons licence will apply to this Work are set out in our licence referred to above.

Other than as permitted in any relevant BMJ Author's Self Archiving Policies, I confirm this Work has not been accepted for publication elsewhere, is not being considered for publication elsewhere and does not duplicate material already published. I confirm all authors consent to publication of this Work and authorise the granting of this licence.

For peer review only

Maternal emotional and physical intimate partner violence and early child development: investigating mediators in a cross sectional study in a South African birth cohort.

Whitney Barnett[a], Sarah L Halligan[b,c], Catherine J Wedderburn[a,c,d,e,], Rae MacGinty[a], Nadia Hoffman[c], Heather J. Zar[a], Dan J. Stein[c,d], Kirsten A Donald[d,f]

[a] Department of Paediatrics and Child Health & South African Medical Research Council Unit on Child & Adolescent Health, University of Cape Town, South Africa; [b] Department of Psychology, University of Bath, United Kingdom; [c] Department of Psychiatry and Mental Health & South African Medical Research Council Unit on Risk and Resilience in Mental Disorders, University of Cape Town, South Africa; [d]Neuroscience Institute, University of Cape Town, South Africa [e]Department of Clinical Research, London School of Hygiene & Tropical Medicine, London, UK; [f] Division of Developmental Paediatrics, Department of Paediatrics & Child Health, University of Cape Town, South Africa

Corresponding author:
Whitney Barnett
Department of Paediatrics and Child Health
Unit B11, REACH Center
Red Cross War Memorial Children's Hospital
Cape Town, South Africa
barnett.whitney@gmail.com
Tel: +27 (0) 790432604

Key words: toddler development, intimate partner violence, LMIC, IPV, child development

Word count: 4,080

Funding
The study was funded by the Bill and Melinda Gates Foundation [OPP 1017641]. Additional support for HJZ and DJS is from the SA MRC. Additional aspects of the work reported here are supported by an Academy of Medical Sciences Newton Advanced Fellowship (NAF002\1001), funded by the UK Government's Newton Fund, by the UK MRC (MR/T002816/1) and by the US Brain and Behaviour Foundation Independent Investigator grant (24467). WB is supported by the SAMRC, through its Division of Research Capacity Development under the Bongani Mayosi National Health Scholars programme. CJW was supported by the Wellcome Trust through a Research Training Fellowship [203525/Z/16/Z].

Acknowledgements

We thank the study staff in Paarl, the study data team and lab teams, the clinical and administrative staff of the Western Cape Government Health Department at Paarl Hospital and at the clinics for support of the study. We thank the families and children who participated in this study.

Abstract

Objectives: This study investigated associations between recent maternal intimate partner violence (emotional, physical and sexual) and child development at 2 years as well as whether maternal depression or alcohol use mediated these relationships.

Design: Cross sectional study nested in a South African birth cohort.

Setting: Two primary care clinics in Paarl, South Africa.

Participants: 626 mother-child pairs; inclusion criteria for maternal antenatal enrolment were clinic attendance and remaining in the study area for at least 1 year; women were excluded if a minor.

Primary outcome measures: Child cognitive, language and motor development composite scores. These were assessed using the Bayley Scales of Infant and Toddler Development, 3rd edition (BSID-III).

Results: Emotional IPV was associated with lower cognitive (β=-0.32; 95%CI -0.60,-0.04), language (β=-0.36; 95%CI -0.69,-0.01) or motor composite scores (β=-0.58; 95%CI -0.95,-0.20) in children at 2 years of age. Physical IPV was associated with lower motor scores (β=-0.42; 95%CI -0.75,-0.09) at 2 years. Sexual IPV was unrelated to developmental outcomes, possibly due to low prevalence. Neither recent maternal depression nor alcohol use were shown to mediate the relationship between IPV and developmental outcomes.

Conclusions: Interventions to reduce maternal physical and emotional IPV and early-life interventions for infants and toddlers are needed to promote optimal child development.

Strengths and limitations of this study

- This study investigates the unique impact of multiple types of IPV (emotional, physical and sexual) on developmental outcomes in very young children in a high-risk setting.
- A key strength of this study includes measurement of a broad range of risk factors and robust measurement of developmental outcomes in a representative sample of participants in a LMIC setting.
- Understanding the factors underlying associations between IPV and poor child development is critical to better inform interventions and policy, thus we investigated both maternal postnatal depression and alcohol use as potential mediators of IPV-child development relationships in this cohort.
- The current study used cross sectional data and therefore did not investigate longitudinal exposures and cannot assert causality.

For peer review only - http://bmjopen.bmj.com/site/about/guidelines.xhtml

Background

Intimate partner violence (IPV) is one of the most common forms of violence against women. The World Health Organization's multicountry study on violence against women found high lifetime prevalence of physical IPV (up to 61%), sexual IPV (up to 59%) and emotional IPV (up to 75%).[1] The epidemic of IPV is particularly marked in low-and-middle-income countries (LMICs), including South Africa, where prevalence is amongst the highest globally.[2,3] Children in households with IPV are often witnesses and are at increased risk for abuse or neglect.[4,5] The United Nations estimates that each year between 133–275 million children witness violence between primary caregivers.[6] Exposure to violence in the home is linked to many adverse health outcomes in children, including developmental, psychological and behavioural impairments.[7-9] Importantly, these often impact health and development beyond childhood, having negative effects that extend into adolescence and adulthood.[8,10] However, there remain several gaps in the existing literature.

Relatively little research has examined associations between the presence of IPV and poorer cognitive, language and motor development in infancy and very early childhood[11-13] though it is a critical developmental period that can have significant implications for long-term health.[14] Where data are available, exposure to IPV in the first two years of life has been associated with increased risk of developmental delay.[12,13] Healthy development during the first years of life is vital, given its impact on academic performance and mental health across the lifespan.[10,14] Indeed, a longitudinal study through 8 years found that exposure to interpersonal trauma (maltreatment and/or witnessing IPV) in the first two years of life was associated with the poorest cognitive outcomes compared to other periods in childhood.[15]

For peer review only

Research focusing on child development and IPV often narrowly defines IPV as only physical or sexual abuse,[12,13,16] though emotional IPV has been linked to similar or increased rates of ill-health, both physical and mental, compared to physical IPV.[17-20] Additionally, the majority of studies, particularly in school-aged children, have been done in high income countries (HICs),[8,11,21] though LMICs have a higher burden of IPV-associated risk factors, such as community violence, trauma, substance use disorders and mental illness and limited support networks. South Africa in particular has among the highest burdens of interpersonal violence in the world.[22] The roots of high levels of violence can be linked to South Africa's colonial and apartheid past, including the impacts of systemic racism, impoverishment, migrant labour, gender norms and constructions of masculinity; their ongoing legacy continues to impact extremely high levels of violence in South Africa.[23] Zembe and colleagues investigated social risk factors and relationship power inequity and IPV in a South African sample, finding high acceptability of violence in intimate relationships as well as gender norms supporting aggressive masculinity and subservient femininity, particularly in historically marginalized groups such as those included in the present study.[24]

Lastly, a key gap in the existing literature examining associations between IPV exposure and early life child developmental outcomes is investigation of potential mediators in this relationship, particularly in LMIC settings. Several potential mechanisms underlying the relationship between IPV and child development have been proposed. For instance, maternal depression as well as alcohol use have been separately linked to both IPV and development outcomes,[13,20] potentially compromising maternal-child attachment, impacting maternal autonomy and caregiving, diverting financial resources in the home and may increase the risk of child maltreatment or abuse.[25] Alcohol is one of the most commonly misused substances, with extremely high

For peer review only - http://bmjopen.bmj.com/site/about/guidelines.xhtml

prevalence of alcohol use disorders in South Africa,[26] and maternal use postnatally is associated with poorer child development and academic performance.[27-29] Maternal depression has been linked to insecure mother-child attachment;[30] less sensitive and responsive caregiving,[31] and reduced health care service utilization[32] adversely impacting care and stimulation needed for healthy development. Although there is evidence linking proposed mediators to both IPV and early-life development, extremely few have formally investigated potential explanatory factors.[33]

This study aims to explore associations between IPV sub-types (emotional, physical and sexual) and child development (cognitive, language and motor) at two years of age and to investigate potential mechanisms underlying this relationship. The current study addresses three key gaps in the literature, specifically by (i) investigating associations between IPV and development in very young children (ii) investigating the differential impact of sub-types of IPV and (iii) investigating maternal depression or alcohol use as potential mediators in these relationships. Given the high prevalence of IPV in low resource settings, understanding the significant impact IPV exposure may have on young children during this developmentally sensitive period and improved understanding of possible pathways underlying these relationships is important for improving child potential globally.[11]

Methods

The parent study is a longitudinal, population-based birth cohort study following mother–child dyads from pregnancy through childhood.[34-36] The study is located in a periurban area in South Africa, characterised by low socioeconomic status (SES) and psychosocial risk factors. There is a free primary health care system, that includes antenatal and child health services. The current cross sectional analysis uses

For peer review only

data from three study visits occurring at or near 2 years of child age; only birthweight and HIV exposure were collected prior to 2 years of age, at birth, as described below.

Participants

Pregnant women were enrolled from March 2012 to March 2015. Women were enrolled in their second trimester, between 20 and 28 weeks' gestation at two public sector primary healthcare clinics, one serving a predominantly mixed-ancestry population and the other serving a predominantly Black African population. Our intention in using the historic terms of *Black African* and *mixed ancestry* is not meant to reify these terms, but to contribute to the literature on ongoing health disparities. Inclusion criteria were: (1) pregnant women attending one of the two study clinics; (2) aged 18 years or older; and (3) intending to remain in the study area for at least 1 year.

Ethical considerations

Ethical approval was obtained from the Faculty of Health Sciences Research Ethics Committee, University of Cape Town (401/2009) and the Western Cape Provincial Research committee. Mothers gave written informed consent at enrolment and were re-consented annually thereafter. Study staff were trained in ethical conduct of violence and mental health research, including confidentiality, and identification and management of risk. Interviews were conducted privately, data were de-identified and only accessible to study staff to ensure confidentiality. Where significant mental health issues or safety concerns were identified, staff referred participants to appropriate care or social services (including support services for IPV, substance abuse and mental health issues).

For peer review only

Clinical and sociodemographic data

Sociodemographic information was collected from the mother at a visit two years after their child's birth, using a standardized questionnaire adapted from items included in the South African Stress and Health Study.[37] Maternal education (any secondary versus completed secondary), employment and partnership status (single or married/marriage-like relationship) and household income [<R,1000/month (60USD), R1,000 to R5000 (350USD) or > R5,000/month] were self-reported at 2 years postpartum. Maternal HIV status during pregnancy was confirmed by routine testing during antenatal care. All HIV-exposed children were tested per the Western Cape prevention of mother-to-child transmission guidelines.[38] Birthweight and postnatal anthropometric measurements were collected by trained staff at birth and 2 years, as has been described.[39]

Intimate partner violence

Recent maternal IPV was measured at 2 years postpartum using the Intimate Partner Violence Questionnaire (IPVQ), a 12-item inventory adapted from the WHO multicountry study[1] and the Women's Health Study in Zimbabwe.[40] The IPVQ assessed recent (past-year) exposure to emotional (4 of 12 questionnaire items), physical (5 of 12 items), and sexual abuse (3 of 12 items). Partner behavior indicating emotional IPV included having been insulted or made to feel bad, having been humiliated in front of others, intentionally scared or intimidated or threatened with physical harm. Physical IPV included being slapped, pushed, shoved, hit with an object, beaten or choked. Sexual IPV exposure was classified based on having been forced to have sex, afraid not to have sex or forced to do something sexual which was degrading or humiliating. Mothers were asked about frequency of exposure to partner behavior

("never", "once", "a few times" or "many times"). Items were then summed to create scores for each IPV sub-type with higher scores indicating greater severity and frequency of exposure; exposure was defined as a score >1 indicating more than an isolated incident within each subtype. Scoring guidelines were devised for the purposes of this study, and were based on prior work in South Africa.[41]

Proposed mediating variables

Recent maternal depression was measured using the Beck Depression Inventory (BDI), a validated and reliable screening tool for depressive symptoms at 2 years post-partum.[42,43] The BDI-II comprises 21 items, which assesses severity of symptoms of major depression on a severity scale from 0 (absence of symptoms) to 3 (severe). Individual items are summed to obtain a total score, with higher scores indicating more severe depressive symptoms. A cut off score of ≥20 was used to indicate participants with probable depression.[44] The Alcohol, Smoking and Substance Involvement Screen Test (ASSIST) was used to measure current maternal alcohol-related risk at 2 years of child age.[45] Alcohol related items were summed to create a score for alcohol-related problems with higher scores indicating greater substance-related risk. We used published guidelines for the ASSIST to classify alcohol scores into three categories for descriptive purposes: low/no risk (0-10), moderate risk (11-26) and high risk (>26), with the likelihood of alcohol dependence.[46]

Bayley Scales of Infant and Toddler Development, 3rd edition (BSID-III)

The Bayley Scales of Infant and Toddler Development, third edition (BSID-III)[47] was used to measure toddler neurodevelopment. It is widely used to assess development in children ages 2 to 42 months and has been validated for use in South

Africa.[48] Assessments for neurodevelopment were done at 2 years of child age by trained physiotherapists and occupational therapists supervised by a paediatric neurodevelopmental specialist.[36] Quality control and monitoring processes were implemented to ensure accuracy. Assessments were conducted with language prompts in the child's preferred language and assessors alternated between sites and assessed a similar number of children. In the current study, composite scores for cognition, language and motor development were used. The composite scores are calculated using a normal US population, and are standardised to have a mean of 100 and standard deviation of 15. Composite scores are strongly correlated to raw scores in the current study and allow comparison across domains. Continuous composite scores as well as categorical outcomes are reported. Categories were defined as 'delay' (<2 standard deviations below mean), 'suboptimal development' (<1 standard deviation below mean) and 'no delay'.

Patient and public involvement

Investigators have established close relationships with key stakeholders including Western Cape Government Health Department and community members. Participants were not involved in the design or recruitment of the DCHS. However, participant experience and satisfaction have been assessed at multiple time points.[49] Participant feedback about study experience, information provided and study procedures have been used ongoing to reassess study protocols and to make alternations to improve study experience and participant satisfaction. Study findings are routinely fed back to the community and the Department of Health clinical and administrative staff.

Data analysis

The analyses were conducted using STATA 15 (StataCorp Inc., College Station, Texas, USA). Demographic, clinical, psychosocial and child development data were described using median (interquartile range (IQR)) for continuous data or number (%) for categorical data. Developmental data were compared by child sex using Pearson's Chi-square test for categorical data and the Wilcoxon Rank Sum test for continuous data.

Mean composite scores were calculated for the Bayley-III for cognitive, language and motor domains. Birthweight was converted to z-scores based on child sex and gestational age using the INTERGROWTH-21st standards [50]. Postnatal weight-for-age z-scores (WFAZ), length-for-age z-scores (LFAZ) and weight-for-length z-scores (WFHZ) were calculated using weight and length measurements at 24 months, using Anthro software (WHO, Geneva, Switzerland) [51]. These as well as emotional, physical and sexual IPV and proposed mediator variables were included as continuous scores in all analyses.

We examined associations using a complete case analysis sample. Bivariate linear regression analyses were used to investigate the relationship between developmental domains, clinical and demographic variables, proposed mediators and IPV sub-types. Regression coefficients with 95% confidence intervals (CIs) are reported. Multivariable linear regression models were then run for each of the developmental (composite cognitive, language and motor scores) outcomes, including significant univariable coefficients ($p<0.05$). Continuous scores were used for exposure, mediator and outcome data in multivariable models. Where multiple IPV sub-types were significant in bivariate regression, adjusted models were run for each separately, as these were highly correlated. Hypothesized mediators of the relationship

For peer review only

between IPV and early life development (current maternal depression and alcohol use) were investigated. Criteria for formal investigation of potential mediators were 1) their association with developmental outcomes, using a cut-off level of $p<0.05$, in final adjusted models (path b) and 2) where associations between IPV and the hypothesized mediator were significant (path a).

In addition to the analysis of observations with complete data, we examined associations after imputing missing data for covariates and developmental outcomes using multiple imputation with chained equations (MICE). We assumed covariate data were missing at random and performed 30 imputations. Results from complete case and imputed datasets produced similar conclusions. Results based on imputed data are presented in Supplemental Tables. To explore potential selection bias, we assessed the characteristics of mother-child dyads with complete and missing data, using Pearson's Chi-squared and Wilcoxon Rank Sum tests to compare covariate distributions between groups.

Results

Of 1143 study births, 154 (13.5%) children were lost to follow up before reaching two years of age primarily due to moving out of the study area or being uncontactable or unavailable for follow up visits. A further 255 (25.8%) did not complete a BSID-III assessment due to non-attendance on the day or moving out of the study area. Data for the current study was collected across three visits at approximately 2 years of child age: a clinical visit for growth and sociodemographic data collection; a psychosocial visit for maternal depression and substance use data and a visit assessing child development. A total of 626 children who completed the BSID-III assessment and had IPV exposure and covariate data were included in the final complete case dataset.

For peer review only - http://bmjopen.bmj.com/site/about/guidelines.xhtml

Comparing the characteristics of dyads with complete and missing exposure or outcome data, mothers with missing data were less likely to be HIV infected (p=0.01) and less likely to be employed (p=0.02, Supplemental Table 1).

Clinical, demographic, and psychosocial characteristics at two years are presented in Table 1. This sample was characterized by low maternal employment (41%), low maternal education (37% completed secondary education), low household income (only 27% earning more than R5,000 per month [approximately USD 350]). Almost half of mothers were married or cohabiting (49%) and 24% of children were HIV exposed with only 2 children HIV-infected. A minority of mothers experienced psychosocial risk factors or substance use, including emotional IPV (16%), physical IPV (17%) and sexual IPV (4%), alcohol (10% moderate/high risk), and depression (6%), Table 1.

Developmental outcomes

A total of 28/625 (4%) children were categorised as having cognitive delay (<2 standard deviations below mean), 48/598 (8%) with language delay and 7/592 (1%) with motor delay. Only language differed significantly by child sex, with males having a higher prevalence of delay compared to females (10% versus 5%; p value=0.001) and lower composite scores (p<0.001), Table 2.

Intimate partner violence and developmental outcomes

At 24 months, bivariate analysis showed that emotional IPV was associated with lower composite cognitive scores (β=-0.32; 95% CI -0.58,-0.06), lower composite language scores (β=-0.35; 95% CI -0.69,-0.01) and lower composite motor scores (β=-0.58; 95% CI -0.93,-0.24). Physical IPV was associated with lower composite motor

view only

scores (β=-0.42; 95% CI -0.73,-0.11). Sexual IPV was not associated with any composite developmental outcomes, Table 3.

Multivariable regression models were run to examine associations between IPV and child developmental outcomes, adjusting for key potential confounding factors. IPV sub-types were run separately in adjusted models due to collinearity and only where significant in bivariate analyses. In adjusted models, the results held, recent emotional IPV was associated with reduced scores across all developmental outcomes, specifically with lower composite cognitive scores (β -0.32; 95% CI -0.60,-0.04), language (β -0.35; 95% CI -0.69, -0.01) and motor scores (β -0.58; 95% CI -0.95, -0.20). Physical IPV was associated with lower motor scores (β -0.42; 95% CI -0.75, -0.09), Table 4. Associations using the imputed dataset were similar for key exposures (IPV) and outcomes (developmental), Supplemental Table 2 & 3.

Hypothesized mediators

Current maternal depression and alcohol use were explored as potential mediators in the relationship between recent IPV sub-types and developmental outcomes. In correlational analyses, both maternal depression and alcohol use were positively associated with emotional as well as physical IPV (Supplemental Table 4). However, when linear regressions were run investigating associations between mediators and developmental outcomes, adjusted for potential confounding factors, we found no evidence of associations between proposed mediators and each developmental outcome (cognitive, language, motor), Supplemental Table 5. Therefore, formal mediation analyses were not done.

For peer review only

Discussion

Our results show that recent maternal emotional IPV was associated with lower cognitive, language and motor scores and physical IPV with lower motor scores in children at 24 months. Further, we investigated the potential role of current maternal depression or alcohol use in this relationship. There was insufficient evidence that current maternal depression or alcohol use were explanatory factors in the relationship between IPV and child development at 2 years of age.

We found associations between emotional IPV and each developmental outcome investigated, which are novel findings in such young children. Though there is limited research in this age group, and existing studies use differing definitions of IPV, a recent study pooling data from 11 LMICs investigating pre-school development (36-59 months) found associations between all IPV sub-types and developmental outcomes.[52] Where investigated in a younger sample, studies have found a link between IPV and early life development. However, few investigated IPV sub-types as separate risk factors for poor development. For example, a US study, investigating the impact of IPV on developmental outcomes at two years reported an increased risk of language and neurological delay in infants and toddlers, however this was not analysed by IPV sub-type but rather a composite measure of emotional, physical and sexual IPV.[12] A Tanzanian study through three years of age found both maternal lifetime physical and lifetime sexual IPV to be associated with reduced motor skills, expressive and receptive communication and cognitive development.[13] Emotional IPV was not included in their IPV measure. One study that did investigate emotional IPV separately as a risk factor for cognitive and language delay, only found associations with postnatal physical IPV and only in unadjusted analyses. The limited sample size (n=72) may have reduced statistical power to detect an association.[53] In the current study, sexual IPV was not

For peer review only

associated in unadjusted or adjusted analyses with the outcomes investigated, but this is likely due to insufficient power to detect an association given the relatively low prevalence of recent sexual IPV in our study population (4%). In the current study, maternal emotional IPV as compared to physical IPV was more consistently associated with poorer developmental outcomes. These results indicate that emotional IPV may carry a unique risk for poor child developmental outcomes in early life in our setting.

Given the high prevalence of emotional IPV and that it is often not as readily recognised or included in research, intervention or policy efforts aimed at prevention of IPV, it is critical to better understand its distinct impact on child health outcomes. Our findings suggest that adverse outcomes and their association with maternal IPV may be underestimated when IPV exposure is not considered broadly and inclusive of emotional IPV, a potentially significant gap in the literature. Emotional abuse in a current or past intimate relationships is increasingly being recognized as a unique contributing factor to maternal ill-health outcomes.[18-20] Specifically, emotional IPV has been linked to poor mental health, post traumatic stress disorder and poorer physical health, sometimes above that of physical IPV.[19,54] Psychological control or abuse by a partner may uniquely impact maternal efficacy, independence and diminish maternal power within the family, which may affect her ability to provide nutritious food, a secure environment or to have positive parenting practices, all of which could lead to increased risk of negative outcomes for her children. These factors may be particularly influential for health outcomes in settings such as South Africa where entrenched gender norms and a high acceptability of violence contribute to high levels of IPV.[24]

Our investigation of potential mediators found little evidence to support that current maternal depression symptoms or reported alcohol use were explanatory factors for the impact of IPV on child development at two years. In the parent study we have

For peer review only

previously found that IPV is linked to longitudinal maternal depression[20] as well as hazardous alcohol use in pregnancy.[55] In the current study, we found that IPV was associated with depression and alcohol use at 2 years. However, these did not appear to be associated with development at 2 years. Recent maternal depression or alcohol use may compromise the quality of parenting or parent-child attachment, may divert household financial resources and decrease maternal care-seeking behaviours, which in turn may compromise child development. Though there is much literature linking maternal substance use to adverse child outcomes, some studies that have investigated its impact on parenting or secure attachment have found mixed results.[56,57] For example, a study assessing maternal substance use and quality of caregiving found no differences between substance abusing and non-substance abusing mothers and caregiving or maternal-child attachment at 12 months.[56] Similarly, studies investigating maternal depression and child development have shown mixed findings.[13,58,59] A Tanzanian study investigating depression and toddler development, found that depression was associated with receptive language and motor development but not cognitive or expressive language.[13] Another study found that maternal depression at 7 weeks postpartum impacted development at 3 months but this relationship attenuated by 6 months.[58] Our findings may be influenced by relatively low levels of probable clinical depression (11%) or moderate-high alcohol-related risk (10%), diluting possible associations. We found no evidence that recent maternal depression or alcohol use influenced the relationship between IPV and early life development; these findings may illustrate the complexity of the relationship between the factors explored and other adverse environmental risks which often co-occur and may be underlying these relationships.

For peer review only

The prospective nature of the parent study, including measurement of a broad range of risk factors and robust measurement of developmental outcomes in an LMIC setting, is a strength of this study. However, there are several limitations. The current study used cross sectional data, therefore we cannot assert causality. Maternal sexual IPV prevalence was very low in our sample (4%) and this likely affected power to discern potential associations. We also did not investigate longitudinal exposures or cumulative impact of maternal depression or alcohol use; these were only investigated as recent exposures. Further, although the BSID-III is a well recognised tool, further standardisation in sub-Saharan African settings is required. For example, the categorisation of delay is based on scaled scores using normative US data that might not be generalisable to a South African population. However, our data from the parent study has shown that raw scores have similar patterns to the results based on delay categorisation. There were some differences in characteristics of those included versus excluded in the present study, namely HIV exposure and maternal employment. Maternal employment was lower in the included sub-sample (41% vs 51%), however, household income was similar across groups. Furthermore, maternal employment was not associated with any outcomes investigated in bivariate analyses and adjustment for this did not alter any of the key associations. HIV exposure was higher in the included sub-sample (24% versus 18%), however, this would likely bolster potential associations. In addition, approximately one-third of children who were still active in the study at two years were not included in the analysis due to incomplete data. However, results were similar when associations were explored in the imputed dataset.

Conclusions

In summary, our results highlight the need for programs that aim to reduce IPV as well as early life prevention efforts to mitigate the impact of maternal IPV on

For peer review only

children. Maternal IPV was a consistent predictor of poor child developmental outcomes; furthermore, emotional IPV emerged as a uniquely deleterious risk factor for child developmental outcomes. Further work is needed to understand the mechanisms underlying this relationship, exploring maternal depression and alcohol use in other samples as well as additional potential mechamisms. Given our findings, it is important for public health professionals and policy makers to ensure that maternal emotional IPV is included in training programs for health care staff as well as in screening and referral efforts. Lastly, more research investigating emotional or psychological IPV and adverse health outcomes is needed. Critically, in our setting, the majority of pregnant and early postpartum women access healthcare, providing an important, potentially under-utilized opportunity to intervene early to attempt to shield children from the negative effects of IPV.

Data sharing

Collaborations for the analysis of data are welcome; the parent study has a large and active group of investigators and postgraduate students and many have successfully partnered with students or researchers from other institutions. Researchers who are interested in datasets or collaborations can find more information on our website [http://www.paediatrics.uct.ac.za/scah/dclhs].

Competing interests

The authors have no conflicts of interest to declare.

Author contributions

WB, KD and SH conceptualized the study aims and methodology. WB with guidance from RM, conducted all analyses. HJZ is principal investigator of the parent study; DS

For peer review only

and NH lead the psychosocial study aspects; CW and KD are coinvestigators and were responsible for implementation, training and quality control of developmental assessments. All authors provided intellectual input and reviewed and approved the final manuscript.

Ethical approval statement

Ethical approval was obtained from the Faculty of Health Sciences Research Ethics Committee, University of Cape Town (401/2009) and the Western Cape Provincial Research committee.

For peer review only

References

1. World Health Organization. 2005. WHO *Multi-country Study on Women's Health and Domestic Violence against Women*. Retrieved 26 April 2021, from https://www.who.int/gender/violence/who_multicountry_study/summary_report/summary_report_English2.pdf.
2. Decker MR, Peitzmeier S, Olumide A, et al. Prevalence and Health Impact of Intimate Partner Violence and Non-partner Sexual Violence Among Female Adolescents Aged 15-19 Years in Vulnerable Urban Environments: A Multi-Country Study. *J Adolesc Health* 2014; **55**(6 Suppl): S58-67.
3. Davis EC, Rotheram-Borus MJ, Weichle TW, Rezai R, Tomlinson M. Patterns of Alcohol Abuse, Depression, and Intimate Partner Violence Among Township Mothers in South Africa Over 5 Years. *AIDS Behav* 2017; **21**(Suppl 2): 174-82.
4. Devries KM, Knight L, Child JC, et al. Witnessing intimate partner violence and child maltreatment in Ugandan children: a cross-sectional survey. *BMJ Open* 2017; **7**(2): e013583.
5. Carlson C, Namy S, Norcini Pala A, et al. Violence against children and intimate partner violence against women: overlap and common contributing factors among caregiver-adolescent dyads. *BMC Public Health* 2020; **20**(1): 124.
6. Pinheiro, P. S. *World Report on Violence Against Children* (United Nations, Geneva, 2006). Retrieved 28 Apr 2020, from https://www.unicef.org/violencestudy/reports/SG_violencestudy_en.pdf.
7. Vu NL, Jouriles EN, McDonald R, Rosenfield D. Children's exposure to intimate partner violence: A meta-analysis of longitudinal associations with child adjustment problems. *Clin Psychol Rev* 2016; **46**: 25-33.
8. Bair-Merritt MH, Blackstone M, Feudtner C. Physical health outcomes of childhood exposure to intimate partner violence: a systematic review. *Pediatrics* 2006; **117**(2): e278-90.
9. Kiesel LR, Piescher KN, Edleson JL. The Relationship Between Child Maltreatment, Intimate Partner Violence Exposure, and Academic Performance. *J Public Child Welf* 2016; **10**(4): 434-56.
10. Gale CR, Cooper R, Craig L, et al. Cognitive function in childhood and lifetime cognitive change in relation to mental wellbeing in four cohorts of older people. *PLoS One* 2012; **7**(9): e44860.
11. Howell KH, Barnes SE, Miller LE, Graham-Bermann SA. Developmental variations in the impact of intimate partner violence exposure during childhood. *J Inj Violence Res* 2016; **8**(1): 43-57.
12. Udo IE, Sharps P, Bronner Y, Hossain MB. Maternal Intimate Partner Violence: Relationships with Language and Neurological Development of Infants and Toddlers. *Matern Child Health J* 2016; **20**(7): 1424-31.
13. Neamah HH, Sudfeld C, McCoy DC, et al. Intimate Partner Violence, Depression, and Child Growth and Development. *Pediatrics* 2018; **142**(1).
14. Shonkoff JP, Garner AS. The lifelong effects of early childhood adversity and toxic stress. *Pediatrics* 2012; **129**(1): e232-46.
15. Enlow MB, Egeland B, Blood EA, Wright RO, Wright RJ. Interpersonal trauma exposure and cognitive development in children to age 8 years: a longitudinal study. *J Epidemiol Community Health* 2012; **66**(11): 1005-10.
16. Kliem S, Kirchmann-Kallas S, Stiller A, Jungmann T. [Impact of Intimate Partner Violence on Child's Cognitive Development - Results from the

Evaluation of the Home Visiting Program "Pro Kind"]. *Prax Kinderpsychol Kinderpsychiatr* 2019; **68**(1): 63-80.
17. Kramer A, Lorenzon D, Mueller G. Prevalence of intimate partner violence and health implications for women using emergency departments and primary care clinics. *Womens Health Issues* 2004; **14**(1): 19-29.
18. Yoshihama M, Horrocks J, Kamano S. The role of emotional abuse in intimate partner violence and health among women in Yokohama, Japan. *Am J Public Health* 2009; **99**(4): 647-53.
19. Coker AL, Davis KE, Arias I, et al. Physical and mental health effects of intimate partner violence for men and women. *Am J Prev Med* 2002; **23**(4): 260-8.
20. Okafor CN, Barnett W, Zar HJ, et al. Associations of Emotional, Physical, or Sexual Intimate Partner Violence and Depression Symptoms Among South African Women in a Prospective Cohort Study. *J Interpers Violence* 2018: 886260518796522.
21. Yount KM, DiGirolamo AM, Ramakrishnan U. Impacts of domestic violence on child growth and nutrition: a conceptual review of the pathways of influence. *Soc Sci Med* 2011; **72**(9): 1534-54.
22. Norman R, Schneider M, Bradshaw D, et al. Interpersonal violence: an important risk factor for disease and injury in South Africa. *Popul Health Metr* 2010; **8**: 32.
23. Coovadia H, Jewkes R, Barron P, Sanders D, McIntyre D. The health and health system of South Africa: historical roots of current public health challenges. *Lancet* 2009; **374**(9692): 817-34.
24. Zembe YZ, Townsend L, Thorson A, Silberschmidt M, Ekstrom AM. Intimate Partner Violence, Relationship Power Inequity and the Role of Sexual and Social Risk Factors in the Production of Violence among Young Women Who Have Multiple Sexual Partners in a Peri-Urban Setting in South Africa. *PLoS One* 2015; **10**(11): e0139430.
25. Atkinson L, Paglia A, Coolbear J, Niccols A, Parker KC, Guger S. Attachment security: a meta-analysis of maternal mental health correlates. *Clin Psychol Rev* 2000; **20**(8): 1019-40.
26. Myers B. LJ, Fakier N. Access to substance abuse treatment in the Cape Town Metropole: Final report. *South African Medical Research Council* 2007.
27. Jester JM, Jacobson SW, Sokol RJ, Tuttle BS, Jacobson JL. The influence of maternal drinking and drug use on the quality of the home environment of school-aged children. *Alcohol Clin Exp Res* 2000; **24**(8): 1187-97.
28. Jansson LM. Maternal Alcohol Use During Lactation and Child Development. *Pediatrics* 2018; **142**(2).
29. Rochat TJ, Houle B, Stein A, Mitchell J, Bland RM. Maternal alcohol use and children's emotional and cognitive outcomes in rural South Africa. *S Afr Med J* 2019; **109**(7): 526-34.
30. Barnes J, Theule J. Maternal depression and infant attachment security: A meta-analysis. *Infant Ment Health J* 2019; **40**(6): 817-34.
31. National Research Council (US) and Institute of Medicine (US) Committee on Depression, Parenting Practices, and the Healthy Development of Children. *Depression in Parents, Parenting, and Children: Opportunities to Improve Identification, Treatment, and Prevention.* 2009. Retrieved 10 Jul 2020, from: https://www.ncbi.nlm.nih.gov/books/NBK215128/.

For peer review only

32. Callo-Quinte G, Del-Ponte B, Ruivo ACO, et al. Maternal depression symptoms and use of child health-care services at The Pelotas 2004 Birth Cohort. *J Affect Disord* 2019; **253**: 303-7.
33. Kitzmann KM, Gaylord NK, Holt AR, Kenny ED. Child witnesses to domestic violence: a meta-analytic review. *J Consult Clin Psychol* 2003; **71**(2): 339-52.
34. Stein DJ, Koen N, Donald KA, et al. Investigating the psychosocial determinants of child health in Africa: The Drakenstein Child Health Study. *J Neurosci Methods* 2015; Aug **30**;252:27-35. doi: 10.1016/j.jneumeth.2015.03.016.
35. Zar HJ, Barnett W, Myer L, Stein DJ, Nicol MP. Investigating the early-life determinants of illness in Africa: the Drakenstein Child Health Study. *Thorax* 2015; **70**(6): 592-4.
36. Donald KA, Hoogenhout M, du Plooy CP, et al. Drakenstein Child Health Study (DCHS): investigating determinants of early child development and cognition. *BMJ Paediatr Open* 2018; **2**(1): e000282.
37. Myer L, Stein DJ, Grimsrud A, Seedat S, Williams DR. Social determinants of psychological distress in a nationally-representative sample of South African adults. *Soc Sci Med* 2008; **66**(8): 1828-40.
38. Pellowski J, Wedderburn C, Stadler JAM, et al. Implementation of prevention of mother-to-child transmission (PMTCT) in South Africa: outcomes from a population-based birth cohort study in Paarl, Western Cape. *BMJ Open* 2019; **9**(12): e033259.
39. Budree S, Stein DJ, Brittain K, et al. Maternal and infant factors had a significant impact on birthweight and longitudinal growth in a South African birth cohort. *Acta Paediatr* 2017; **106**(11): 1793-801.
40. Shamu S, Abrahams N, Zarowsky C, Shefer T, Temmerman M. Intimate partner violence during pregnancy in Zimbabwe: a cross-sectional study of prevalence, predictors and associations with HIV. *Trop Med Int Health* 2013; **18**(6): 696-711.
41. Dunkle KL, Jewkes RK, Brown HC, Gray GE, McIntryre JA, Harlow SD. Gender-based violence, relationship power, and risk of HIV infection in women attending antenatal clinics in South Africa. *Lancet* 2004.
42. Beck AT, Steer RA, Carbin MG. Psychometric properties of the Beck Depression Inventory: Twenty-five years of evaluation. *Clin Psychol Rev* 1988; **8**(1): 77-100.
43. Kagee A, Nel A, Saal W. Factor structure of the Beck Depression Inventory-II among South Africans receiving antiretroviral therapy. *AIDS Care* 2014; **26**(2): 257-62.
44. Lasa L, Ayuso-Mateos JL, Vázquez-Barquero JL, Díez-Manrique FJ, Dowrick CF. The use of the Beck Depression Inventory to screen for depression in the general population: a preliminary analysis. *J Affect Disord* 2000; **57**(1-3): 261-5.
45. Humeniuk R, Ali R, Babor TF, et al. Validation of the Alcohol, Smoking And Substance Involvement Screening Test (ASSIST). *Addiction* 2008; **103**(6): 1039-47.
46. World Health Organization. 2010. *The Alcohol, Smoking and Substance Involvement Screening Test (ASSIST) Manual for use in primary care.* Retrieved 28 June 2020, from http://www.who.int/substance_abuse/publications/assist/en/.

For peer review only

47. Bayley N. Pearson; Bloomington, MN: 2006. Bayley scales of infant and toddler development, 3rd edition-technical manual.
48. Rademeyer V, Jacklin L. A study to evaluate the performance of black South African urban infants on the Bayley Scales of Infant Development III. *SA J Child Health* 2013; **7**: 54-9.
49. Barnett W, Brittain K, Sorsdahl K, Zar HJ, Stein DJ. Maternal participant experience in a South African birth cohort study enrolling healthy pregnant women and their infants. *Philos Ethics Humanit Med* 2016; **11**(1): 3.
50. Villar J, Papageorghiou AT, Pang R, et al. The likeness of fetal growth and newborn size across non-isolated populations in the INTERGROWTH-21st Project: the Fetal Growth Longitudinal Study and Newborn Cross-Sectional Study. *Lancet Diabetes Endocrinol* 2014; **2**(10): 781-92.
51. World Health Organization. 2006. *'WHO Child Growth Standards: length/height-for-age, weight-for-age, weight-for-length, weight-for-height and body mass index-for-age: methods and development.* Geneva: World Health Organization. Acta Paediatr Suppl, 2006; 450: 76–85.52.
52. Jeong J, Adhia A, Bhatia A, McCoy DC, Yousafzai AK. Intimate Partner Violence, Maternal and Paternal Parenting, and Early Child Development. *Pediatrics* 2020; **145**(6): e20192955.
53. Rodriguez VJ, Peltzer K, Matseke G, Weiss SM, Shine A, Jones DL. Pre- and postnatal exposure to intimate partner violence among South African HIV-infected mothers and infant developmental functioning at 12 months of age. *Arch Womens Ment Health* 2018; **21**(6): 707-13.
54. Tiwari A, Chan K, Fong D, et al. The impact of psychological abuse by an intimate partner on the mental health of pregnant women. *Bjog* 2008; **115**(3): 377-84.
55. Myers B, Koen N, Donald KA, et al. Effect of Hazardous Alcohol Use During Pregnancy on Growth Outcomes at Birth: Findings from a South African Cohort Study. *Alcohol Clin Exp Res* 2018; **42**(2): 369-77.
56. Bergin C, McCollough P. Attachment in substance-exposed toddlers: The role of caregiving and exposure. *Infant Ment Health J* 2009; **30**(4): 407-23.
57. Eiden RD, Edwards EP, Leonard KE. Mother-infant and father-infant attachment among alcoholic families. *Dev Psychopathol* 2002; **14**(2): 253-78.
58. Galler JR, Harrison RH, Ramsey F, Forde V, Butler SC. Maternal depressive symptoms affect infant cognitive development in Barbados. *J Child Psychol Psychiatry* 2000; **41**(6): 747-57.
59. Morais MdLSe, Lucci TK, Otta E. Postpartum depression and child development in first year of life. *Estudos de Psicologia (Campinas)* 2013; **30**: 7-17.

For peer review only

Table 1. Sample maternal and child psychosocial, demographic and clinical characteristics, n=626*

	Total *n=626*
Intimate Partner Violence (24 months)	
Emotional score, median (IQR)	0 (0, 0)
Emotional (above threshold), n (%)	102 (16)
Physical score, median (IQR)	0 (0, 0)
Physical (above threshold), n (%)	107 (17)
Sexual score, median (IQR)	0 (0, 0)
Sexual (above threshold), n (%)	25 (4)
Any sub-type score, median (IQR)	0 (0,0)
Any sub-type (above threshold), n (%)	138 (22)
Sociodemographics (24 months)	
Maternal education (secondary not completed), n (%)	233 (37)
Mother employed, n (%)	225/545 (41)
Mother married/partnered, n (%)	268/544 (49)
Household Income	
<R5,000 (approximately 300 USD), n (%)	445/609 (73)
>R5,000, n (%)	164/609 (27)
Recruitment site: TC Newman, n (%)	274 (44)
Physical variables	
Birth weight z-score, median (IQR)**	-0.59 (-1.34, 0.16)
Child age at assessment (months), median (IQR)	24.05 (23.79, 24.31)
HIV exposed**, n (%)	153 (24)
Length for age z-score at 24 months, median (IQR)	-1.16 (-1.89, -0.52)
Weight for age z-score at 24 months, median (IQR)	-0.31 (-1.10, 0.43)
Weight for length z-score at 24 months, median (IQR)	0.28 (-0.48, 1.21)
Psychosocial variables (24 months)	
Depression score, median (IQR)	0 (0, 3)
Depression (above threshold), n (%)	36/591 (6)
Alcohol dependence score, median (IQR)	0 (0, 0)
Alcohol dependence (categories)	
Low dependence, n (%)	160/593 (27)
Moderate dependence, n (%)	33/593 (6)
High dependence, n (%)	22/593 (4)

Notes: Median (IQR) ranges reported for anthropometry data, intimate partner violence scores as well as depression and alcohol scores. Frequencies (percentages) reported for all other variables. All percentages calculated on non-missing values. N values are indicated where the number of participants with available data differs from the total group (n=626). Intimate partner violence, above threshold exposure defined as more than an isolated incident within each sub-type. Depression threshold defined as BDI score of >=20. Alcohol dependence categories defined as: low/no risk (0-10), moderate risk (11-26) and high risk (>26), with the likelihood of alcohol dependence.
*Includes data from mothers or children included in complete case dataset.
**Birthweight and HIV exposure collected at birth; all other variables collected at 2 years of child age.

For peer review only

Table 2. Developmental outcomes of children at 2 years of age by child sex

	Total	Female	Male	p-value
Sample	*n=625*	*n=303*	*n=322*	
Cognitive scores, mean (SD)	85.74 (8.84)	86.44 (8.56)	85.08 (9.06)	0.035
Cognitive categories				
No delay, n (%)	424 (68)	218 (72)	206 (64)	
Suboptimal development, n (%)	173 (28)	75 (25)	98 (30)	
Delayed, n (%)	28 (4)	10 (3)	18 (6)	0.078
Sample	*n=598*	*n=289*	*n=309*	
Language scores, mean (SD)	84.45 (11.39)	86.57 (11.35)	82.47 (11.08)	<0.001
Language categories				
No delay, n (%)	297 (50)	164 (57)	133 (43)	
Suboptimal development, n (%)	253 (42)	110 (38)	143 (46)	
Delayed, n (%)	48 (8)	15 (5)	33 (11)	0.001
Sample	*n=592*	*n=289*	*n=303*	
Motor scores, mean (SD)	93.62 (11.89)	94.40 (12.12)	92.86 (11.63)	0.111
Motor categories				
No delay, n (%)	490 (83)	243 (84)	247 (82)	
Suboptimal development, n (%)	95 (16)	41 (14)	54 (18)	
Delayed, n (%)	7 (1)	5 (2)	2 (1)	0.251

Continuous composite scores are presented as mean (SD). Categorical data presented as frequencies (percentages). Categories were defined as ‘delay’ (<2 standard deviations below mean), ‘suboptimal development’ (<1 standard deviation below mean) and ‘no delay’. P-values presented are comparing sex differences.

For peer review only

Table 3. Univariate associations between intimate partner violence, covariates and proposed mediators and composite scores for developmental domains in complete case analysis.

	**Composite Cognitive (n=549)**	**Composite Language (n=598)**	**Composite Motor (n=521)**
	Unadjusted	Unadjusted	Unadjusted
	Coefficient (95% CI)	Coefficient (95% CI)	Coefficient (95% CI)
Intimate Partner Violence (IPV)			
Emotional IPV score	-0.32 (-0.58, -0.06)*	-0.35 (-0.69, -0.01)*	-0.58 (-0.93, -0.24)*
Physical IPV score	-0.18 (-0.42, 0.05)	-0.23 (-0.54, 0.08)	-0.42 (-0.73, -0.11)*
Sexual IPV score	-0.15 (-0.97, 0.67)	-0.16 (-1.29, 0.97)	-0.49 (-1.63, 0.65)
Sociodemographics			
Maternal education (not completed secondary)	-1.31 (-2.70, 0.08)	-2.86 (-4.61, -1.11)*	-0.93 (-2.78, 0.92)
Mother employed	0.60 (-0.87, 2.08)	0.34 (-1.57, 2.27)	0.68 (-1.29, 2.65)
Mother married/partnered	-0.52 (-1.98, 0.93)	0.47 (-1.43, 2.37)	0.01 (-1.94, 1.95)
Household Income			
>R5000	0.73 (-0.92, 2.38)	0.32 (-1.80, 2.45)	1.63 (-0.55, 3.82)
Recruitment site: TC Newman	1.19 (-0.16, 2.53)	3.05 (1.35, 4.76)**	1.13 (-0.66, 2.93)
Physical variables			
Birth weight z-score	0.42 (-0.17, 1.01)	0.84 (0.09, 1.58)*	0.62 (-0.17, 1.39)
Male	-1.66 (-3.01, -0.32)*	-4.27 (-5.96, -2.59)**	-1.52 (-3.31, 0.27)
Child age (months)	-0.94 (-2.23, 0.34)	-0.10 (-1.74, 1.53)	-2.81 (-4.49, -1.12)*
HIV exposed	-1.71 (-3.30, -0.11)*	-3.90 (-5.93, -1.87)**	-0.76 (-2.89, 1.38)
Length for age z-score	0.97 (0.36, 1.58)*	0.77 (-0.01, 1.55)	0.13 (-0.70, 0.97)
Weight for age z-score	0.92 (0.32, 1.51)*	0.64 (-0.12, 1.40)	0.68 (-0.19, 1.47)
Weight for length z-score	0.61 (0.02, 1.19)*	-0.34 (-0.41, 1.09)	0.84 (0.05, 1.63)*
Proposed Psychosocial mediators			
Depression score	-0.02 (-0.11, 0.08)	-0.06 (-0.18, 0.06)	-0.12 (-0.25, 0.01)
Alcohol dependence score	-0.07 (-0.17, 0.03)	-0.03 (-0.16, 0.11)	0.01 (-0.13, 0.15)

Notes: All variables measured at 2 years of child age except birth weight, sex and HIV-exposure which were captured at birth. IPV sub-types, depression and alcohol dependence were included as continuous variables in all models.
Abbreviations: IPV=intimate partner violence.
*p<0.05; **p<0.001;

For peer review only

Table 4. Multivariable linear regression demonstrating the adjusted association of intimate partner violence and composite scores for developmental domains in complete case analysis sample.

	**Composite Cognitive (n=549)**	**Composite Language (n=598)**	**Composite Motor (n=521)**	
	Adjusted# Coefficient (95% CI)	Adjusted^ Coefficient (95% CI)	Adjusted+ Coefficient (95% CI)	Adjusted+ Coefficient (95% CI)
Emotional IPV score	-0.32 (-0.60, -0.04)*	-0.35 (-0.69, -0.01)*	-0.58 (-0.95, -0.20)*	
Physical IPV score				-0.42 (-0.75, -0.09)*

Adjusted models were run only where IPV sub-type was associated with outcome explored ($p<0.05$) in bivariate analyses. For cognitive and language scores, only emotional IPV was associated in bivariate analyses and therefore run in multivariable models. Due to collinearity, emotional and physical IPV were run in separate multivariable models for motor development. IPV sub-types, depression and alcohol dependence were included as continuous variables in all models.
Abbreviations: IPV=intimate partner violence.
*p<0.05; **p<0.001
#Adjusted for child sex, HIV exposure, length-for-age z-scores, weight-for-age z-scores and weight-for-length z-scores at 24 months.
^Adjusted for maternal education, recruitment site, weight-for-age z-score at birth, child sex and HIV exposure.
+Adjusted for child age at assessment and weight-for-length z-scores at 24 months.

For peer review only

Supplemental Table 1. Comparison of psychosocial, demographic and clinical data between children included in complete case developmental models and those who are not.

	In Development models	Not in Development model	p-value
Intimate Partner Violence (IPV)			
Emotional IPV score, median (IQR)	0 (0, 0)	0 (0, 0)	0.669
Physical IPV score, median (IQR)	0 (0, 0)	0 (0, 0)	0.213
Sexual IPV score, median (IQR)	0 (0, 0)	0 (0, 0)	0.160.
Sociodemographics			
Maternal education (did not complete secondary)	393 (63)	302 (58)	0.132
Mother employed	225 (41)	99 (51)	0.022*
Mother married/partnered	268 (49)	99 (51)	0.718
Household Income			
<R5000	445 (73)	122 (63)	0.671
>R5000	164 (27)	47 (24)	
Recruitment site: TC Newman	274 (44)	235 (45)	0.586
Physical variables			
Birth weight z-score, median (IQR)	-0.59 (-1.34, 0.16)	-0.51 (-1.22, -0.00)	0.744
Male sex	321 (51)	269 (52)	0.774
HIV exposed	153 (24)	94 (18)	0.010*
Length for age z-scores, median (IQR)	-1.10 (-1.80, -0.34)	-1.03 (-1.85, -0.28)	0.846
Weight for age z-scores, median (IQR)	-0.31 (-1.10, 0.43)	-0.24 (-1.01, 0.40)	0.6682
Weight for length z-scores, median (IQR)	0.28 (-0.48, 1.13)	0.43 (-0.40, 1.09)	0.7542
Psychosocial factors			
Depression score, median (IQR)	0 (0, 3)	0 (0, 5)	0.415
Alcohol dependence score, median (IQR)	0 (0,0)	0 (0,0)	0.068

Data are presented as n(%) or median IQR). Birthweight and HIV exposure collected at birth; all other variables collected at 2 years of child age. Abbreviation: IPV=intimate partner violence.
*p<0.05; **p<0.001

For peer review only

Supplemental Table 2. Univariate associations between intimate partner violence, covariates and proposed mediators and composite scores for developmental domains in the multiple-imputation analysis sample.

	Composite Cognitive	Composite Language	Composite Motor
	Unadjusted	Unadjusted	Unadjusted
	Coefficient (95% CI)	Coefficient (95% CI)	Coefficient (95% CI)
Intimate Partner Violence (IPV)			
Emotional IPV score	-0.33 (-0.60, -0.06)*	-0.34 (-0.68, -0.01)*	-0.51 (-0.86, -0.16)*
Physical IPV score	-0.19 (-0.43, 0.05)	-0.20 (-0.50, 0.11)	-0.39 (-0.70, -0.09)*
Sexual IPV score	-0.13 (-0.98, 0.71)	-0.16 (-1.36, 1.04)	-0.41 (-1.58, 0.75)
Sociodemographics			
Maternal education (not completed secondary)	-1.31 (-2.69, 0.07)	-3.20 (-4.94, -1.45)**	-1.45 (-3.30, 0.40)
Mother employed	0.48 (-1.02, 1.99)	0.13 (-1.81, 2.07)	0.41 (-1.55, 2.38)
Mother married/partnered	-0.59 (-2.07, 0.89)	0.26 (-1.64, 2.16)	0.15 (-1.88, 2.18)
Household Income			
>R5000	0.73 (-0.92, 2.38)	1.00 (-1.14, 3.13)	1.96 (-0.28, 4.20)
TC Newman	1.20 (-0.15, 2.54)	3.12 (1.41, 4.84)**	0.89 (-0.91, 2.68)
Physical variables			
Birth weight z-score	0.44 (-0.15, 1.03)	0.68 (-0.07, 1.42)	0.58 (-0.21, 1.36)
Male	-1.67 (-3.01, -0.33)*	-4.07 (-5.77, -2.37)**	-1.70 (-3.49, 0.09)
Child age (months)	-0.95 (-2.24, 0.33)	-0.12 (-1.77, 1.53)	-2.81 (-4.50, -1.11)*
HIV exposed	-1.70 (-3.29, -0.10)*	-3.77 (-5.82, -1.72)**	-0.59 (-2.72, 1.54)
Length for age z-score	0.92 (0.33, 1.52)*	0.67 (-0.09, 1.42)	0.26 (-0.54, 1.06)
Weight for age z-score	0.84 (0.26, 1.43)*	0.64 (-0.10, 1.37)	0.82 (0.04, 1.60)*
Weight for length z-score	0.50 (-0.08, 1.08)	0.39 (-0.34, 1.11)	0.88 (0.10, 1.66)*
Proposed Psychosocial mediators			
Depression score	-0.02 (-0.12, 0.08)	-0.06 (-0.18, 0.07)	-0.11 (-0.24, 0.02)
Alcohol dependence score	-0.08 (-0.19, 0.03)	-0.03 (-0.17, 0.11)	0.03 (-0.11, 0.16)

Birthweight and HIV exposure collected at birth; all other variables collected at 2 years of child age. IPV sub-types, depression and alcohol dependence were included as continuous variables in all models.
Abbreviation: IPV= intimate partner violence.
*p<0.05, **p<0.001

Supplemental Table 3. Multivariable linear regression demonstrating the association of intimate partner violence with composite scores for developmental domains in the multiple-imputation analysis sample.

	Cognitive scores	Language scores	Motor scores	
	Adjusted#	Adjusted^	Adjusted+	Adjusted+
	Coefficient (95% CI)	Coefficient (95% CI)	Coefficient (95% CI)	Coefficient (95% CI)
Emotional IPV score	-0.30 (-0.57, -0.03)*	-0.35 (-0.68, -0.01)	-0.52 (-0.87, -0.17)*	
Physical IPV score				-0.39 (-0.70, -0.09)*

Adjusted models were run only where IPV sub-type was associated (p<0.05) with outcome explored in bivariate analyses. For cognitive and language scores, only emotional IPV was associated in bivariate analyses and therefore run in multivariable models. Due to collinearity, emotional and physical IPV were run in separate multivariable models for motor development. Adjusted models were run including same covariates as the complete case analysis, as noted below. IPV sub-types, depression and alcohol dependence were included as continuous variables in all models.
Abbreviations: IPV=intimate partner violence.
*p<0.05; **p<0.001
#Adjusted for child sex, HIV exposure, length-for-age z-scores, weight-for-age z-scores and weight-for-length z-scores at 24 months.
^Adjusted for maternal education, recruitment site, weight-for-age z-score at birth, child sex and HIV exposure.
+Adjusted for child age at assessment and weight-for-length z-scores at 24 months.

For peer review only

Supplemental Table 4. Univariate associations between proposed mediators and intimate partner violence in complete case sample.

	**Emotional IPV** Coefficient (95% CI)	**Physical IPV** Coefficient (95% CI)
Depression score	1.01 (0.80, 1.22)**	0.68 (0.49, 0.87)**
Alcohol dependence score	0.34 (0.13, 0.56)*	0.30 (0.12, 0.49)*

IPV sub-types, depression and alcohol dependence were included as continuous variables in all models.
Abbreviation: IPV = intimate partner violence.
*p<0.05, **p<0.001

Supplemental Table 5. Univariate and adjusted associations between proposed mediators and developmental outcomes in complete case sample.

		**Cognitive scores**# Coefficient (95% CI)	**Language scores**^ Coefficient (95% CI)	**Motor scores**+ Coefficient (95% CI)
Univariate	Depression	-0.02 (-0.11, 0.08)	-0.06 (-0.18, 0.06)	-0.12 (-0.25, 0.01)
	Alcohol	-0.07 (-0.17, 0.03)	-0.03 (-0.16, 0.11)	0.01 (-0.13, 0.15)
Adjusted	Depression	0.02 (-0.08, 0.13)	-0.05 (-0.18, 0.07)	-0.04 (-0.19, 0.10)
	Alcohol	-0.06 (-0.17, 0.05)	0.01 (-0.12, 0.14)	0.05 (-0.10, 0.20)

Notes: Adjusted associations between proposed mediators and developmental domains were run using linear regression. Depression and alcohol were run separately in adjusted regression models. IPV sub-types, depression and alcohol dependence were included as continuous variables in all models.
Abbreviation: IPV = intimate partner violence.
#Adjusted for intimate partner violence, child sex, HIV exposure, length-for-age z-scores, weight-for-age z-scores and weight-for-length z-scores at 24 months.
^Adjusted for intimate partner violence, maternal education, recruitment site, weight-for-age z-score at birth, child sex and HIV exposure.
+Adjusted for intimate partner violence, child age at assessment and weight-for-length z-scores at 24 months.

For peer review only

STROBE Statement—Checklist of items that should be included in reports of ***cohort studies***

	Item No	Recommendation	Page No
**Title and abstract**	1	(*a*) Indicate the study's design with a commonly used term in the title or the abstract	1
		(*b*) Provide in the abstract an informative and balanced summary of what was done and what was found	2
**Introduction**			
Background/rationale	2	Explain the scientific background and rationale for the investigation being reported	3-5
Objectives	3	State specific objectives, including any prespecified hypotheses	5
**Methods**			
Study design	4	Present key elements of study design early in the paper	5
Setting	5	Describe the setting, locations, and relevant dates, including periods of recruitment, exposure, follow-up, and data collection	5/6
Participants	6	(*a*) Give the eligibility criteria, and the sources and methods of selection of participants. Describe methods of follow-up	6
		(*b*) For matched studies, give matching criteria and number of exposed and unexposed	NA
Variables	7	Clearly define all outcomes, exposures, predictors, potential confounders, and effect modifiers. Give diagnostic criteria, if applicable	6-9
Data sources/ measurement	8*	For each variable of interest, give sources of data and details of methods of assessment (measurement). Describe comparability of assessment methods if there is more than one group	6-9
Bias	9	Describe any efforts to address potential sources of bias	11
Study size	10	Explain how the study size was arrived at	11
Quantitative variables	11	Explain how quantitative variables were handled in the analyses. If applicable, describe which groupings were chosen and why	10-11
Statistical methods	12	(*a*) Describe all statistical methods, including those used to control for confounding	10-11
		(*b*) Describe any methods used to examine subgroups and interactions	N/A
		(*c*) Explain how missing data were addressed	Supplemental
		(*d*) If applicable, explain how loss to follow-up was addressed	
		(*e*) Describe any sensitivity analyses	Supplemental
**Results**			
Participants	13*	(a) Report numbers of individuals at each stage of study—eg numbers potentially eligible, examined for eligibility, confirmed eligible, included in the study, completing follow-up, and analysed	11
		(b) Give reasons for non-participation at each stage	11
		(c) Consider use of a flow diagram	
Descriptive data	14*	(a) Give characteristics of study participants (eg demographic, clinical, social) and information on exposures and potential confounders	12 Table 1
		(b) Indicate number of participants with missing data for each variable of interest	
		(c) Summarise follow-up time (eg, average and total amount)	
Outcome data	15*	Report numbers of outcome events or summary measures over time	12, Table 2

For peer review only

For peer review only - http://bmjopen.bmj.com/site/about/guidelines.xhtml

Main results	16	(*a*) Give unadjusted estimates and, if applicable, confounder-adjusted estimates and their precision (eg, 95% confidence interval). Make clear which confounders were adjusted for and why they were included<br>(*b*) Report category boundaries when continuous variables were categorized<br>(*c*) If relevant, consider translating estimates of relative risk into absolute risk for a meaningful time period	12-13<br>Tables 3,4<br><br>7-8<br>Tables 1,2
Other analyses	17	Report other analyses done—eg analyses of subgroups and interactions, and sensitivity analyses	Supplemental tables
**Discussion**			
Key results	18	Summarise key results with reference to study objectives	13-14
Limitations	19	Discuss limitations of the study, taking into account sources of potential bias or imprecision. Discuss both direction and magnitude of any potential bias	16-17
Interpretation	20	Give a cautious overall interpretation of results considering objectives, limitations, multiplicity of analyses, results from similar studies, and other relevant evidence	17
Generalisability	21	Discuss the generalisability (external validity) of the study results	16-17
**Other information**			
Funding	22	Give the source of funding and the role of the funders for the present study and, if applicable, for the original study on which the present article is based	1

*Give information separately for exposed and unexposed groups.

Note: An Explanation and Elaboration article discusses each checklist item and gives methodological background and published examples of transparent reporting. The STROBE checklist is best used in conjunction with this article (freely available on the Web sites of PLoS Medicine at http://www.plosmedicine.org/, Annals of Internal Medicine at http://www.annals.org/, and Epidemiology at http://www.epidem.com/). Information on the STROBE Initiative is available at http://www.strobe-statement.org.

For peer review only

For peer review only - http://bmjopen.bmj.com/site/about/guidelines.xhtml
